# Gene Acquisition Convergence between Entomopoxviruses and Baculoviruses

**DOI:** 10.3390/v7041960

**Published:** 2015-04-13

**Authors:** Julien Thézé, Jun Takatsuka, Madoka Nakai, Basil Arif, Elisabeth A. Herniou

**Affiliations:** 1 Institut de Recherche sur la Biologie de l’Insecte, CNRS UMR 7261, Université François-Rabelais, UFR Sciences et Techniques, 37200 Tours, France; E-Mail: theze.julien@gmail.com; 2 Université de Poitiers, UMR CNRS 7267 Ecologie et Biologie des Interactions, Equipe Ecologie Evolution Symbiose, 86073 Poitiers, France; 3 Forestry and Forest Products Research Institute, Matsunosato, Tsukuba, Ibaraki 305-8687, Japan; E-Mail: junsan@ffpri.affrc.go.jp; 4 Institute of Agriculture, Tokyo University of Agriculture and Technology, Saiwai, Fuchu, Tokyo 183-8509, Japan; E-Mail: madoka@cc.tuat.ac.jp; 5 Great Lakes Forestry Centre, Sault Sainte Marie, ON P6A 2E5, Canada; E-Mail: basil.arif@nrcan-rncan.gc.ca

**Keywords:** genomic convergence, horizontal gene transfer, viral adaptation, insect large DNA viruses

## Abstract

Organisms from diverse phylogenetic origins can thrive within the same ecological niches. They might be induced to evolve convergent adaptations in response to a similar landscape of selective pressures. Their genomes should bear the signature of this process. The study of unrelated virus lineages infecting the same host panels guarantees a clear identification of phyletically independent convergent adaptation. Here, we investigate the evolutionary history of genes in the accessory genome shared by unrelated insect large dsDNA viruses: the entomopoxviruses (EPVs, *Poxviridae*) and the baculoviruses (BVs). EPVs and BVs have overlapping ecological niches and have independently evolved similar infection processes. They are, in theory, subjected to the same selective pressures from their host’s immune responses. Their accessory genomes might, therefore, bear analogous genomic signatures of convergent adaption and could point out key genomic mechanisms of adaptation hitherto undetected in viruses. We uncovered 32 homologous, yet independent acquisitions of genes originating from insect hosts, different eukaryotes, bacteria and viruses. We showed different evolutionary levels of gene acquisition convergence in these viruses, underlining a continuous evolutionary process. We found both recent and ancient gene acquisitions possibly involved to the adaptation to both specific and distantly related hosts. Multidirectional and multipartite gene exchange networks appear to constantly drive exogenous gene assimilations, bringing key adaptive innovations and shaping the life histories of large DNA viruses. This evolutionary process might lead to genome level adaptive convergence.

## 1. Introduction

All cellular and viral species are engaged in multitrophic interactions involving a wide diversity of beneficial or antagonist partners [1]. It is therefore not uncommon for many parasites, including viruses, of unrelated families to thrive within the same host species [2]. In theory, sympatric parasites subjected to similar ecological landscapes (*i.e.*, host immune responses) may select similar mutations to adapt to their hosts [3,4]. The strongest evidence of adaptation though is the convergent evolution in independent lineages of similar traits and genomic features [5,6].

Involved in antagonistic yet durable interactions with their hosts, viruses are strongly locked in a coevolutionary fitness race [7], requiring rapid and efficient genomic innovations. Large dsDNA viruses with their relatively fluid genomes provide sufficient genomic information to test if adaptation towards the same hosts might have produced similar traces on their genomes, particularly the acquisition of beneficial genes [8]. So far, most studies have focused on the convergent acquisition of specific beneficial genes between phylogenetically related viruses infecting Bacteria [9,10]. However, convergence at the genomic level is rarely demonstrated because phylogenetic independence is seldom achieved.

For pathogens, insects are taxonomically the most diverse group of potential mutlicellular eukaryotic host species [11]. Interest in insect pest pathogens for biocontrol applications [12] generated an abundance of viral genomic data. The *Entomopoxvirinae* (EPV) a sub-family of the *Poxviridae* and the family *Baculoviridae* (BV) are large dsDNA viruses exclusively pathogenic to insects [13]. Similar in size, EPVs and BVs, respectively, encode 250–350 and 100–200 genes on 230–330 and 80–180 kb genomes [13,14]. However, their divergent genomic structure (linear *versus* circular) and gene content indicate the virus families are phylogenetically unrelated, and probably have different origins with no common ancestors [15]. The EPV and BV core genomes, containing 49 and 37 essential genes [16,17], only overlap for the *DNA polymerase*, sustaining the replication of their DNA genomes. The remaining genes found in EPV and BV genomes have not been inherited from the origin of either families and form the so-called accessory genomes [18]. EPV and BV share a number of homologous accessory genes [19], including some similar to cellular genes possibly acquired from horizontal gene transfers (HGTs) [20,21,22,23]. Locked in a coevolutionary arms-race, it is presumed that viruses retain only the genes that increase their fitness [8] in a selective trade-off between adaptive novelties and efficient genome replication. This indicates the adaptive role of accessory genomes. EPVs and BVs have largely overlapping ecological niches and can infect the same host species. They might therefore bear analogous signatures of their adaption to these hosts immune response. Furthermore, they have independently evolved a similar infection process. The onset of infections occurs after the ingestion by insect larvae of virus particles, embedded within non-homologous occlusion bodies (OBs), which confer some protection against inactivating environmental factors such as heat, desiccation and UV light [24].

Here we investigated the convergent evolution of the accessory genome content of unrelated large dsDNA viruses based on four recently available EPV genomes [14], infecting the same moths as previously sequenced BVs. This guarantees a clear phyletically independent identification of common genomic features. Furthermore, we estimated the putative phyletic origin of shared accessory genes to determine whether particular gene acquisition processes might be favored. Finally, we discuss the potential role played by mobile elements in gene exchange networks.

## 2. Materials and Methods

### 2.1. Viral Orthologous Clustering

An in-house bioinformatic pipeline was written to cluster together homologous EPV and BV proteins and to search for cellular and viral homologs of each EPV/BV clusters. First, we performed a protein secondary structure alignment clustering, using the jackhmmer program of the hmmer3 package [25], in an all-against-all alignment (threshold: e-value = 10^−6^) of all protein members of *Entomopoxvirinae* sub-family and *Baculoviridae* family found in the non-redundant protein database of the NCBI, including the newly described *Adoxophyes honmai* EPV (AHEV), *Choristoneura biennis* EPV (CBEV), *Choristoneura rosaceana* EPV (CREV) and *Mythimna separata* (MySEV) predicted proteins [14]. Then, with the ClustalOmega program [26], protein multiple alignments were conducted on clusters shared by both virus families, prior to hidden Markov chain (HMM) profile building. By using hmmsearch program of the hmmer3 package, HMM profiles were aligned to the non-redundant protein database and to the insect EST and TSA databases of the NCBI, both of which were initially converted into protein sequences using the metagenomic option of Prodigal program [27]. Finally, for each sequence found, we searched for taxonomic information and selected six categories: (1) other insect large dsDNA (including nudiviruses, ascoviruses, iridoviruses and hytrosaviruses); (2) non-insect large dsDNA viruses; (3) invertebrates; (4) vertebrates; (5) plants’ and (6) bacteria. If needed, we limited the results to the top 20 matches for each category for efficient phylogenetic reconstruction.

### 2.2. Phylogenetic Analyses

Maximum likelihood (ML) phylogenetic inferences were performed on each HMM profile and its associated matches, after conversion into protein multiple alignments. ML analyses were performed with RAxML 7.4.2 [28], using the substitution model and model parameters Whelan And Goldman model with Gamma distribution (WAG+G) and support for node in ML trees were obtained from 100 bootstrap iterations.

## 3. Results

### 3.1. Entomopoxviruses and Baculoviruses Share Accessory Genes

We developed a pipeline, to first cluster all available EPV and BV proteins by secondary structure alignment and to align them against different NCBI databases. Out of 487 viral protein clusters, we found 33 clusters of homologous genes shared by EPVs and BVs (blastp e-value threshold: 1e-6), including 26 with similarities to cellular proteins and 21 with similarities to other families of large dsDNA viruses (Table 1). As expected, we found only one core gene, the *DNA polymerase*, among these homologous clusters. The remaining genes were from the accessory genomes. Several genes encoded essential cellular enzymes such as: the Cu/Zn superoxide dismutase, the ribonucleotide reductase small subunit, the dUTPase, the ubiquitin and the matrixin metalloproteinase. We identified genes potentially involved in insect immune interactions including two families of apoptosis inhibitors (BV *iap3* and *iap4* and *p35/p49*), two kinds of *protein tyrosine phosphatase*, conotoxins and a phage antirepressor. We found multicopy viral gene families, like the *bro* (baculovirus repeated ORFs), MTG (myeloid translocation gene) motif, leucine rich, and nla (nuclear localization of G-actin). Furthermore, we detected two genes related to host ecology: the *fusolin* and the *DNA photolyase*. The remaining genes had previously been described as unknown BV genes.

### 3.2. Viruses Infecting the Same Hosts Share Homologous Genes

Four EPVs in our dataset had overlapping host species with BVs, allowing the investigation of gene convergence in sympatry. The cluster “unknown XecnGV orf138” (*xc138*) was the only one to show substantially higher sequence similarities (Table 1, e-value = 0) between an EPV, MySEV and a BV, *Xestia c-nigrum* granulovirus (XecnGV) (92% over 650 aa), than between XecnGV and its close relative *Pseudaletia unipuncta* granulovirus (PsunGV) (71% over 650 aa). Singularly, these three viruses are able to infect the host *Mythimna separata* [29] as well as a number of other noctuid moths. Pseudogenized forms of *xc138* were detected in the genome of *Helicoverpa armigera* granulovirus (HearGV), which is a sister species of XecnGV, and in the genome of *Heliothis virescens* ascovirus 3g (*Ascoviridae*, HvAV-3g). Interestingly both viruses can infect *M. separata* in the lab though they have only been isolated from *H. armigera* and *Spodoptera exigua*, respectively. More distant baculovirus *xc138* homologs were also identified in *Chrysodeixis chalcites* nucleopolyhedrovirus and *Orgyia leucostigma* nucleopolyhedrovirus, and a second copy in XecnGV.

The acquisition of a *xc138* gene copy might confer to MySEV and XecnGV specific virulence towards their hosts, including for example *M. separata*. In different ecological contexts, as in HearGV, which is more specific to *H. armigera*, the removal of particular host pressure could lead to gene loss in a fitness trade-off between genome replication efficacy and host adaptation. We first hypothesized that both EPVs and BVs might have independently acquired the *xc138* gene from their hosts. However, we could not detect any *xc138* cellular homolog either by bioinformatics data mining or by PCR from *M. separata*, *M. unipuncta*, *M. riparia*, *Xestia c-nigrum*, *Trichoplusia ni*, *Chrysodeixis chalcites* or *Agrotis segetum* genomic DNA. In contrast, we were able to amplify a *xc138* fragment in a closely related EPV of MySEV: *Heliothis armigera* entomopoxvirus (HAEV) (Genbank accession number: KJ683043).

**Table 1:** Origin of homologous gene clusters found in EPVs and BVs.

Viral homologous gene cluster name	Best blastp hit between EPVs and BVs (e-value)	Presence in other large dsDNA viruses	Putative origins of EPV/BV genes	Phylogeny (Figure)
DNA polymerase	6 × 10^−9^	X	-	-
bro gene family protein	9 × 10^−26^	X	Bacteria	S1
helicase 2	1 × 10^−67^	X	Bacteria	S2
matrixin metalloproteinase	1 × 10^−9^	X	Bacteria	S3
nla gene he65 (AcMNPV orf105)	8 × 10^−97^	-	Bacteria	S4
protein tyrosine phosphatase 2 (ptp-2)	2 × 10^−33^	X	Bacteria	S5
putative phage antirepressor	9 × 10^−26^	X	Bacteria	S6
unknown LdMNPV orf129	2 × 10^−57^	-	Bacteria	S7
chitin binding protein (AcMNPV orf145)	1 × 10^−17^	X	Insecta	S8
Cu/Zn superoxide dismutase	4 × 10^−56^	X	Insecta	2b/S9
inhibitor of apoptosis	4 × 10^−79^	X	Insecta	S10
MTG motif gene family protein	7 × 10^−10^	X	Insecta	S11
ubiquitin	6 × 10^−38^	X	Insecta	S12
unknown AcMNPV orf7	5 × 10^−9^	-	Insecta	S13
unknown XecnGV orf106	3 × 10^−13^	X	Insecta	S14
unknown XecnGV orf22	6 × 10^−103^	X	Insecta	S15
acetyltransferase	7 × 10^−13^	-	Insecta (Lepidoptera)	S16
protein phosphatase 1, regulary subunit 15A	5 × 10^−15^	X	Insecta (Lepidoptera)	S17
ribonucleotide reductase small subunit homolog	8 × 10^−134^	X	Insecta (Lepidoptera)	2a/S18
unknown XecnGV orf72	8 × 10^−133^	-	Insecta (Lepidoptera)	S19
protein tyrosine phosphatase 1 (ptp-1)	4 × 10^−41^	X	Insecta (Lepidoptera) & Eukaryote	S20
DNA photolyase	3 × 10^−140^	X	Bilateria/Insecta (Lepidoptera)	S21
dUTPase	1 × 10^−43^	X	Insecta/Unknown	S22
unknown ClanGV orf085	9 × 10^−17^	X	Eukaryote	S23
leucine rich gene family protein	4 × 10^−27^	-	Unicellular eukaryote	S24
fusolin/spindlin/gp37 (AcMNPV orf64)	4 × 10^−67^	-	Unicellular eukaryote (Amoebozoa)	2c/S25
conotoxin-like protein	3 × 10^−20^	-	Virus	S26
p35/p49 apoptosis inhibitor	4 × 10^−30^	-	Virus	S27
unknown AcMNPV orf18	8 × 10^−11^	-	Virus	S28
unknown AdorNPV orf110	8 × 10^−11^	X	Virus	S29
unknown AgseGV orf4	7 × 10^−103^	-	Virus	NA
unknown ChocGV orf11	3 × 10^−14^	-	Virus	NA
unknown XecnGV orf138	0	X	Virus	1a

**Figure 1:**
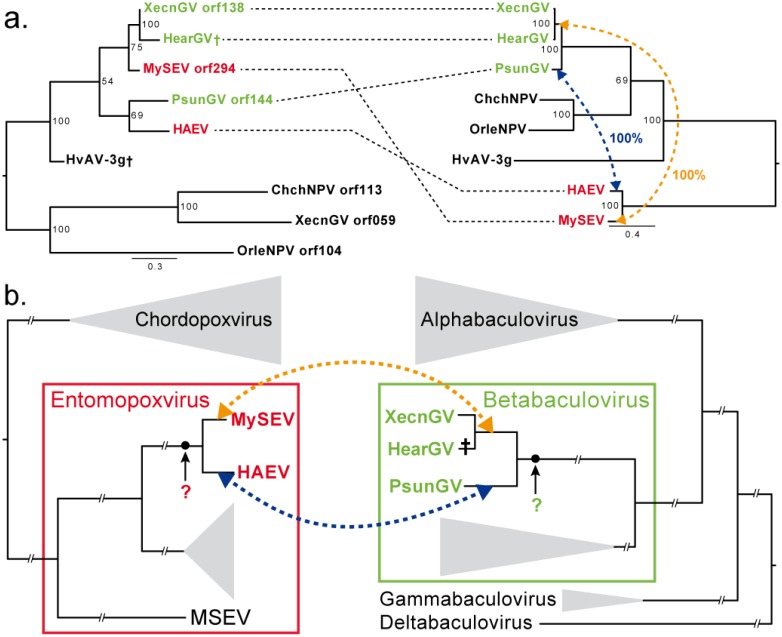
Acquisition convergence of the gene *xc138*. (**a**) Phylogenetic reconciliation between *xc138* gene tree (**left**) and *DNA polymerase* gene tree, representing the virus species tree (**right**) shows two horizontal gene transfers between *Mythimna separata* entomopoxvirus (MySEV) and the *Xestia c-nigrum* granulovirus (XecnGV)/*Helicoverpa armigera* granulovirus (HearGV) common ancestor (in orange) and between *Heliothis armigera* entomopoxvirus (HAEV) and *Pseudaletia unipuncta* granulovirus (PsunGV) (in blue), both with high confidence supports (100%); (**b**) Schematic phylogenies of *Poxviridae* and *Baculoviridae* families show the most parsimonious evolutionary history of *xc138* gene. The black circles denote the acquisition of the *xc138* gene by the entomopoxvirus or baculovirus lineages from an unknown origin and later the two recent horizontal gene transfers between entomopoxviruses and baculoviruses.

The comparison of the *DNA polymerase* phylogeny, representing the virus species tree, with the *xc138* phylogeny highlighted two *xc138* HGTs have occurred between EPVs and BVs (Figure 1a). Reconciliation analyses by bipartition dissimilarity [30] proposed that *xc138* was transferred once between MySEV and the ancestor of XecnGV and HearGV, and a second time between HAEV and PsunGV (Figure 1a,b). The direction of HGTs between these EPVs and BVs is unknown because the presence of a *xc138* gene copy in the common ancestors of both EPV and BV lineages cannot be established. However, this suggests unrelated viruses, such as XecnGV and MySEV, sharing the same hosts can exchange specific genes such as *xc138*. Its subsequent loss in HearGV, suggest *xc138* might be involved in viral adaptation to particular ecological context and convergently maintained in the genomes of XecnGV and MySEV.

### 3.3. Convergence between Viruses Infecting Different Hosts

At the evolutionary timescale, gene acquisition convergence could point out adaptations linked to essential conserved virus-host interactions. We performed maximum likelihood phylogenetic inferences on each of the 31 remaining protein clusters shared between EPVs and BVs (Table 1, Supporting information Figures S1–S29). Unexpectedly, we found 15 genes had putative insect origins, suggesting they were derived from HGTs from host genomes to the virus genomes. Three genes had putative eukaryotic origin outside of the metazoa and seven putatively derived from bacteria, suggesting viruses could have acquired them from the environment or from co-infecting agents. Seven genes, including *xc138*, had no cellular homologs, and were, therefore, assigned a putative viral origin.

**Figure 2:**
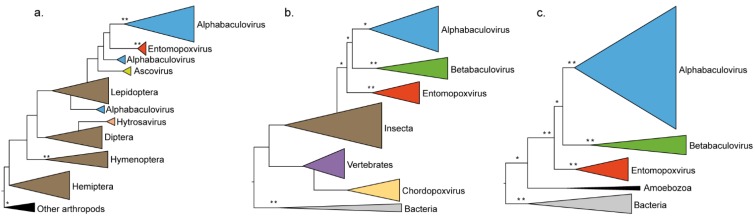
Schematic phylogenies of *rr2*, *sod* and *fusolin* genes. The trees were obtained from maximum likelihood inferences of the multiple amino acid alignments of (**a**) the *rr2*; (**b**) the *sod* and (**c**) the *fusolin* gene products. Support for nodes above branches indicate maximum likelihood nonparametric bootstraps (100 replicates). Bootstraps values between 50 and 75 and upper to 75 are denoted with * and ** respectively.

The *ribonucleotide reductase small subunit* phylogeny (*rr2*, Figure 2a and Supporting information Figure S18) shows the paraphyly of the insects with multiple inclusions of lepidopteran virus clades (BVs, EPVs and ascoviruses) in the Lepidoptera clade. This implies independent and relatively recent *rr2* HGTs from diverse moth genomes to various large DNA viruses, suggesting convergent adaptive acquisitions of host genes across several unrelated virus families infecting a spectrum of related hosts.

Similarly, the *Cu/Zn-superoxide dismutase* phylogeny (*sod*, Figure 2b and Supporting information Figure S9) shows the inclusion of lepidopteran EPV and BV lineages in the insect *sod* clade, and thus the convergent adaptive acquisitions of insect *sod* genes by EPVs and BVs. Moreover, within the BV clade, the *sod* phylogeny reflects the *Baculoviridae* phylogeny [13]. This implies the BV *sod* gene was acquired from an ancestral insect *sod* gene early in baculovirus history, and possibly as early as 90 million years ago [31]. During this time viral sequences have greatly diverged from host sequences, which might explain the low bootstrap support we could recover for the deepest nodes of the tree.

Strikingly both the *rr2* and *sod* phylogeny also reveal higher levels of convergent adaptive gene acquisitions. The inclusions of a dipteran virus clade (*Hytrosaviridae* family) within the Diptera clade in the *rr2* tree (Figure 2a and Supporting information Figure S18) and of the *Chordopoxvirus* (ChPV) clade within the vertebrate lineage in the *sod* tree (Figure 2b and Supporting information Figure S9) indicate convergent gene acquisitions can be independent of the type of host. Both *rr2* and *sod* genes have been recurrently acquired from their hosts by unrelated viruses infecting both insects and vertebrates. This is strong evidence of viral adaptive convergence towards essential cellular processes encountered in all metazoans.

The *fusolin* phylogeny (Figure 2c and Supporting information Figure S25) shows convergent ancestral acquisitions of the *fusolin* gene occurred early in the evolution of both EPVs and BVs, as it reflects both the *Entomopoxvirinae* [14] and *Baculoviridae* phylogenies [13]. The *fusolin* appears absent in metazoans and its closest cellular relatives are chitin-binding proteins of amoeba and bacteria. This suggests EPVs and BVs have captured the *fusolin* from an alternate cellular organism than their natural insect hosts.

## 4. Discussion

Previous studies on genomic convergent evolution highlighted similar mutations and HGTs outcomes for bacteria sharing particular ecological niches [3,4]. Phyletic constraint could have imposed convergent adaptation on the bacteria, due to their shared history as cellular organisms. As viruses have multiple independent origins [32], we studied unrelated virus lineages infecting the same panel of hosts to identify phyletically independent convergent adaptations underpinned by key genomic adaptive mechanisms, as yet undetected in eukaryotic viruses. Although they are phylogenetically unrelated, EPVs and BVs share remarkably similar replication strategies. Both EPVs and BVs produce pseudo-crystalline OBs for *per os* infection of insect larvae as well as spindle bodies enhancing the initial stages of midgut infection [24]. As this phenotypic convergence should be traceable at the genomic level, we looked for homologous genes between EPVs and BVs and investigated their evolutionary history using phylogenetic analyses.

We identified 32 clusters of homologous genes present in the accessory genomes of both EPVs and BVs (Table 1) signaling substantial convergent gene acquisitions. Strikingly, the *xc138* gene of XecnGV is 92% identical to that of MySEV, suggesting a recent HGT between these virus lineages (Figure 1b). Notably, the presence of *xc138* might be correlated with virus host range, as both MySEV and XecnGV are able to infect *M. separata*. Close homologs, recently acquired by other EPVs, BVs and ascoviruses, might be implicated in specific host interactions as all infect closely related noctuid host species. However these five viruses carrying *xc138* are all able to infect a larger range of host species than most of EPVs and BVs that do not carry this gene. Thus, this gene might alternatively confer a wider host range. Further functional analyses are however necessary to specify the phenotype linked with this gene.

Besides these recent HGTs, we found gene acquisition convergence anchored deeper in time, early in the history of virus families, as illustrated by the *rr2*, *sod* and *fusolin* phylogenies (Figure 2). In particular, the *sod* phylogeny (Figure 2b) underlines ancestral gene acquisition convergence between unrelated virus families towards insects, but also within the *Poxviridae* between two virus lineages of non-overlapping host spectrum (vertebrates and insects). Markedly, phylogenetic analyses on the origin of common EPV and BV accessory genes show HGT from insect hosts to be the most common source of exogenous gene assimilations (Table 1). But viruses appear to also have acquired genes from cellular organisms other than their natural hosts. This implies that eukaryotic large DNA viruses could undergo HGTs all across the tree of life [33].

Despite extensive effort to find cellular homologs, some genes seem exclusively viral (Table 1). Their presence, in independent phylogenetic lineages, shows gene exchanges could occur between viruses. For example the *xc138* and *fusolin* trees (Figure 1 and Figure 2c) showed HGTs from EPVs to BVs. In the case of the *fusolin*, codon bias studies had suggested the HGT [34]. Furthermore, in EPVs the product of the *fusolin* are spindle bodies, which can enhance BV *per oral* infectivity [35]. This implies the immediate adaptive benefit BV could have gained from the *fusolin* HGT from an EPV. Apparently absent from metazoans, the *fusolin* closest cellular relatives are chitin-binding proteins of amoeba and bacteria. Thus EPVs might have captured an ancestral form of the *fusolin* from other cellular organisms than their natural hosts. The window for such a gene capture is necessarily smaller than for a gene from a natural host, reducing the opportunity for recurrent gene transfer into different virus families. Consequently, the opportunity to acquire this gene from a cellular organism might have happened only once thus in only one virus family. *Fusolin* confers a significant adaptive advantage in *per oral* infectivity [36], which is the natural route of infection for both EPVs and BVs. Transmission of an ancestral *fusolin* gene from EPVs to BVs could have occurred afterwards, but still early in the evolution of both groups, allowing the BV lineage to adapt more efficiently to lepidopteran hosts. Even if gene transfers between viruses are possible, we posit that they are relatively rare events in comparison with HGT from hosts, which are the majority in our dataset. The EPV/BV models are particularly appropriate for observing these outstanding exchanges, as they have similar infection processes and mixed infections of the same hosts occur [37,38]. Though replicating in different cell compartments (BVs in the nucleus and EPVs in the cytoplasm), mixed infections of the same cells could support viral gene exchanges, although the molecular mechanisms remain elusive.

**Figure 3:**
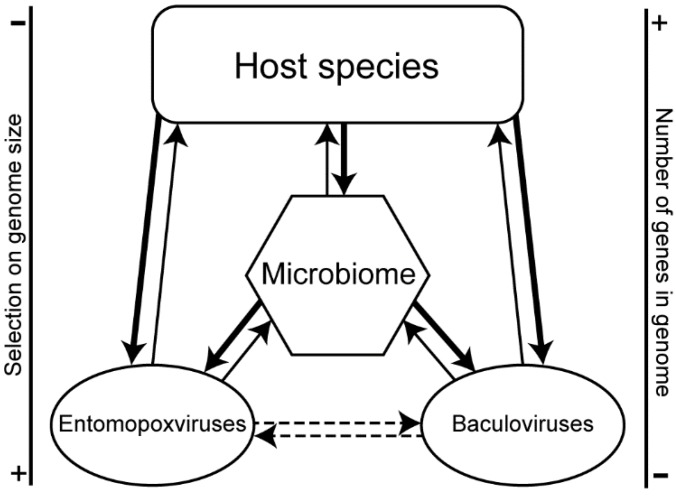
Hologenome gene exchange networks. Many routes of horizontal gene transfers within the hologenome of host species and its interacting partners can lead to gene acquisition convergence between entomopoxviruses and baculoviruses. The thickness of arrows indicates the occurrence of horizontal gene transfers (bold: very frequent, regular: frequent) and dotted arrows indicate possible gene exchanges.

Gene acquisition seems to be a widespread phenomenon in large dsDNA viruses. However, it is presumed that viruses retain only the genes that increase their fitness. So during the course of evolutionary history, genes have profusely been captured and lost. Only those genes conferring substantial adaptive benefits could spread within virus populations through natural selection. Previous studies have shown different gene exchanges involving virus, multicellular eukaryotes and unicellular organisms. Gene transfers can occur from viruses to multicellular eukaryote hosts, as in the case of endogenous viral elements [39] and by whole genome integration, as in polydnaviruses [6]. Recurrent HGTs have also been shown between bacteria and multicellular eukaryote hosts [40] and between viruses and bacteria [41]. As viruses interact with other viruses and cellular organisms, within what makes up the host hologenome [42], each interacting partner becomes a potential source of genes. We propose that these hologenomes support networks of multipartite and multidirectional gene exchanges (Figure 3) providing a large reservoir of new potentially adaptive genes to all partners including viruses.

Several families of transposable elements integrate into BV genomes during infection cycles and are thus present at low frequency within BV populations [43]. It is possible that other large DNA viruses, such as EPV could also be the targets of transposable elements. Moreover, the discovery of new mobile elements has recently soared and their implication in genome evolution seems substantial [44,45]. The genomes of these mobile elements can include genes from various origins (eukaryotic, bacterial and viral) that could have been acquired from host genomes as well as from microbial genomes. These mobile elements spreading within the genomes and populations of various organisms, including viruses [46], could be assimilated afterwards, allowing the delivery of horizontally transferred genes. Although research on these elements remains in its infancy, they could possibly facilitate gene exchanges during coinfections between viruses replicating in different cellular compartments, such as EPVs and BVs, and thus participate in the evolution of gene contents within hologenomes.

Depending on their phylogenetic origin, the genes of large dsDNA virus are broadly classified as belonging to the essential core genome or to the accessory genome. By containing beneficial non-essential genes, the accessory genome is in essence more plastic than the core genome and is derived principally from the assimilation (acquisition and adaptive molecular evolution) of exogenous genes. Viruses probably rely on this accessory gene reservoir to adapt to ecological niches. The signatures of adaptive evolution should be obvious in the accessory genome. Accessory genes have been found to undergo episodes of positive selection [21], duplication and loss events [47] that participate in virus genome dynamics. We find that unrelated large dsDNA viruses infecting closely or distantly related hosts converge towards homologous genomic adaptation. This demonstrates adaptive gene assimilation is a general evolutionary process. Our study based on EPVs and BVs with unbalanced numbers of sequenced genomes (~60 for BVs and only 6 for EPVs), identified 32 homologous genes between EPVs and BVs (Table 1), which could perform analogous adaptive functions. The facts that 19 of these genes were found in other large dsDNA virus families, including the chordopoxviruses, nudiviruses, ascoviruses, iridoviruses and hytrosaviruses further emphasize the recurrence of adaptive convergence and the fundamental influence of ecological constraints on genome evolution.

We observed adaptive HGTs throughout the evolutionary history of insect viruses. Some early HGTs are phylogenetically conserved after assimilation and might have been instrumental in overcoming particularly complex infections barriers, such as host innate immunity. The production of superoxide [48] and induction of apoptosis are innate host mechanisms that control viral infection [49]. The recurrent convergent acquisitions and evolutionary maintenance of the *sod* and antiapoptotic genes (*iap* and *p35*) [50] in viruses are evidence of the evolutionary leaps that follow adaptive gene assimilation. Other genes such as the different *ribonucleotide reductase* subunits or the *dUTPase* are periodically acquired and lost, showing their importance for viral replication success might depend more on specific environmental host conditions. The similarity in host ecology also drives the accessory genome convergence, as in the case of *fusolin*, enhancing *per oral* infectivity.

Our definition of networks of multipartite and multidirectional gene exchanges supports that the accessory genomes of large dsDNA virus can be considered as collections of gene acquired from cells and other viruses, and which can be expanded via intragenomic duplications. The consequence is an entanglement of the origins of viral genes. This could explain why, so far, the evolution of the first viruses remains mysterious. Closer to us, seeking adaptive gene convergence in viruses adapted to specific host species could bring new developments in virus biocontrol and in vaccines applications. HGTs and convergence in cellular and viral gene acquisition gave major adaptive impulses to large dsDNA virus evolution, fostering evolutionary leaps in virus life histories.

## Supplementary Files

Supplementary File 1
