# Supplementary material for: Gene Acquisition Convergence between Entomopoxviruses and Baculoviruses
Source: Viruses. 2015 Apr 13;7(4):1960–74. doi: 10.3390/v7041960 (PMC4411684; doi:10.3390/v7041960)
Supplement: Supplementary File 1 [file viruses-07-01960-s001.pdf]

Supporting Information

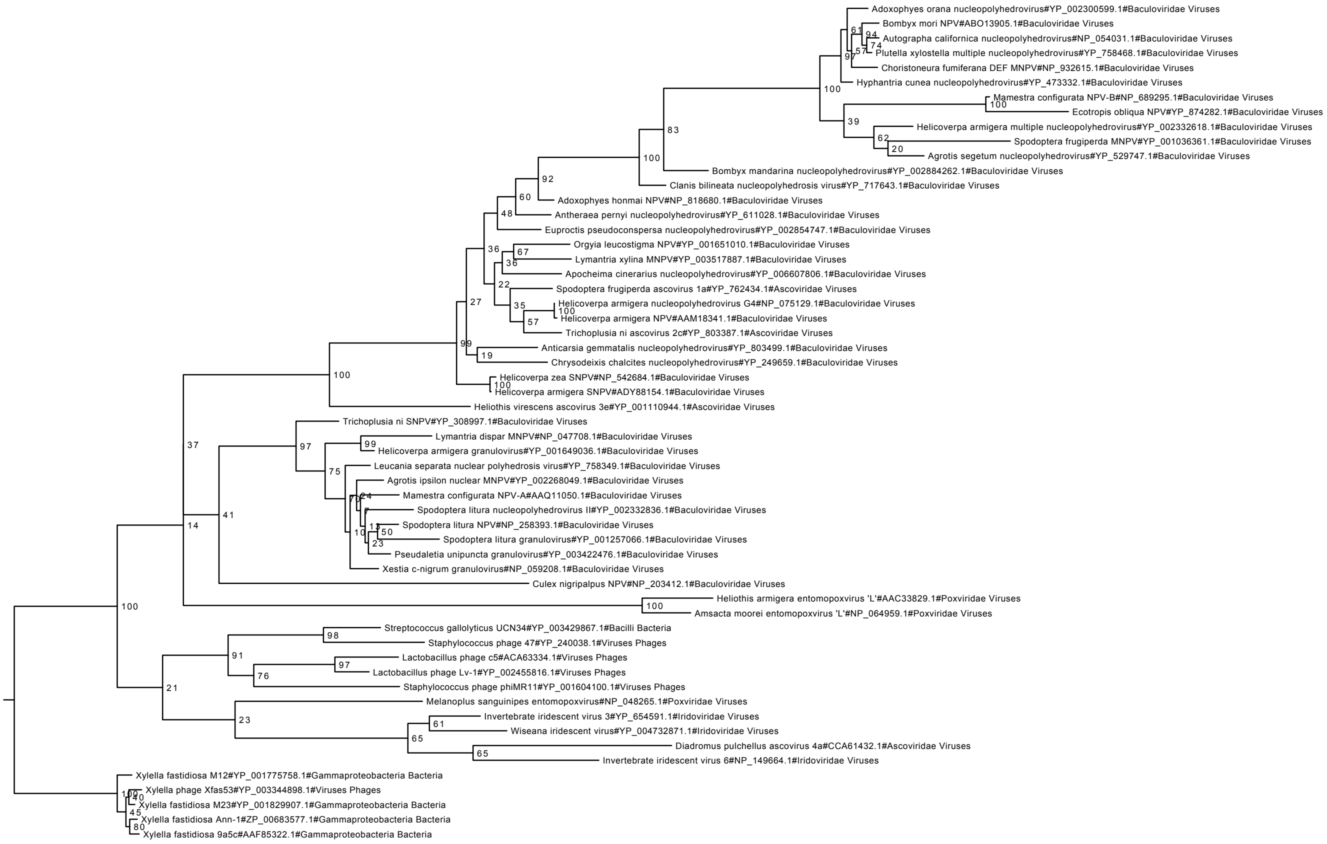

Figure S1. bro gene family protein phylogeny. The tree was obtained from a maximum likelihood inference of the bro gene family protein multiple amino acid alignments, including homologues found in baculoviruses and poxviruses, in other large DNA viruses and in cellular organisms. Support for nodes indicate maximum likelihood nonparametric bootstraps (100 replicates).

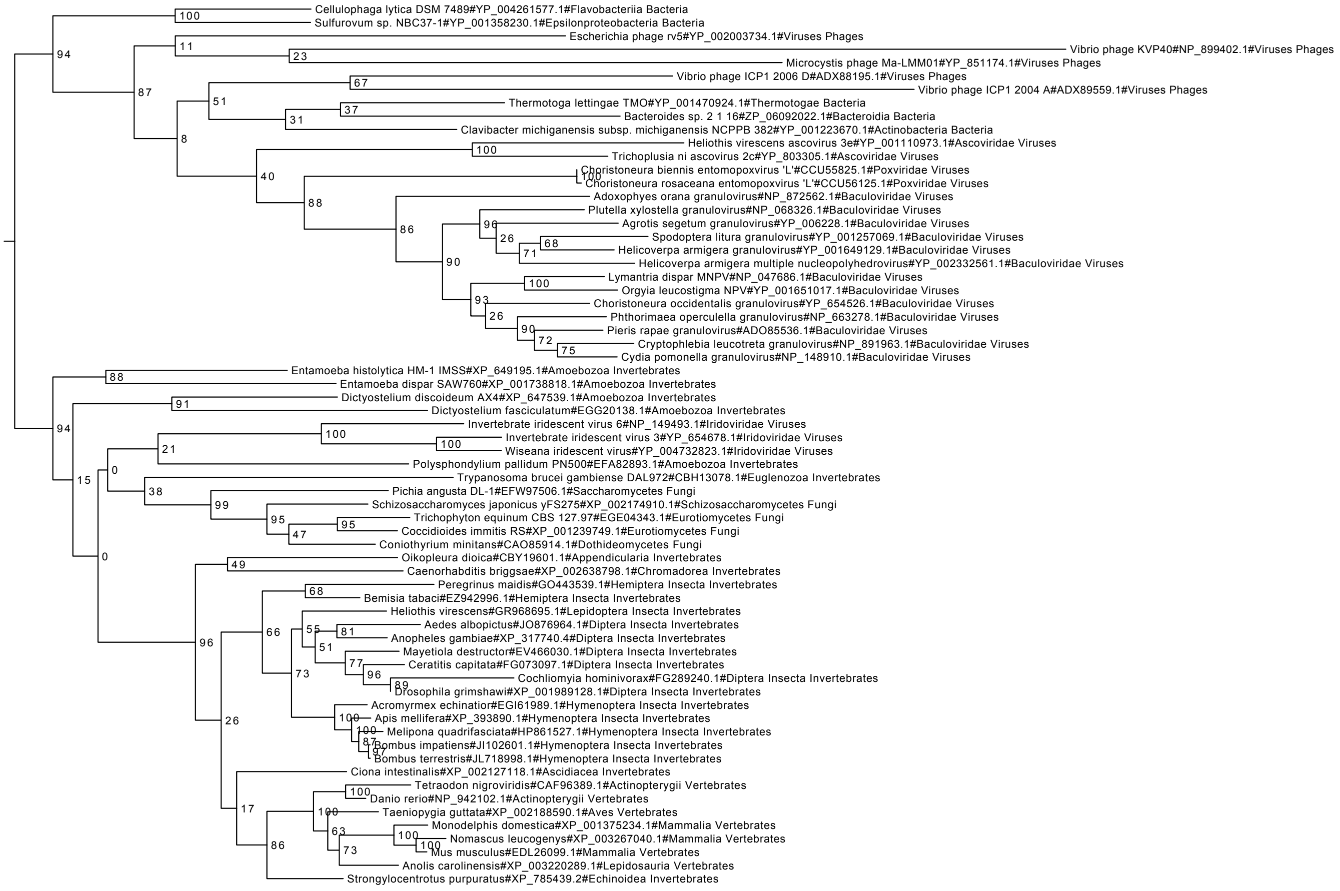

Figure S2. helicase 2 phylogeny. The tree was obtained from a maximum likelihood inference of the helicase 2 multiple amino acid alignments, including homologues found in baculoviruses and poxviruses, in other large DNA viruses and in cellular organisms. Support for nodes indicate maximum likelihood nonparametric bootstraps (100 replicates).

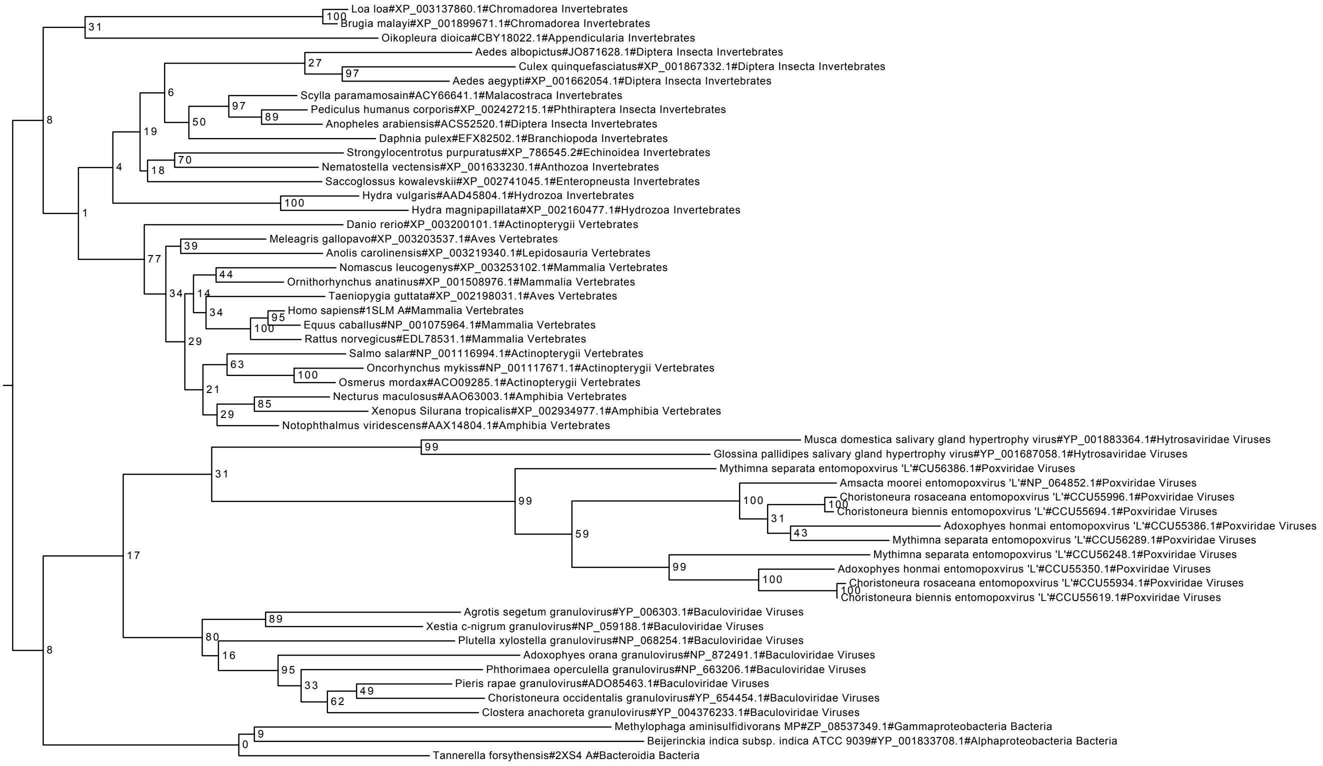

Figure S3. matrixin metalloproteinase phylogeny. The tree was obtained from a maximum likelihood inference of the matrixin metalloproteinase multiple amino acid alignments, including homologues found in baculoviruses and poxviruses, in other large DNA viruses and in cellular organisms. Support for nodes indicate maximum likelihood nonparametric bootstraps (100 replicates).

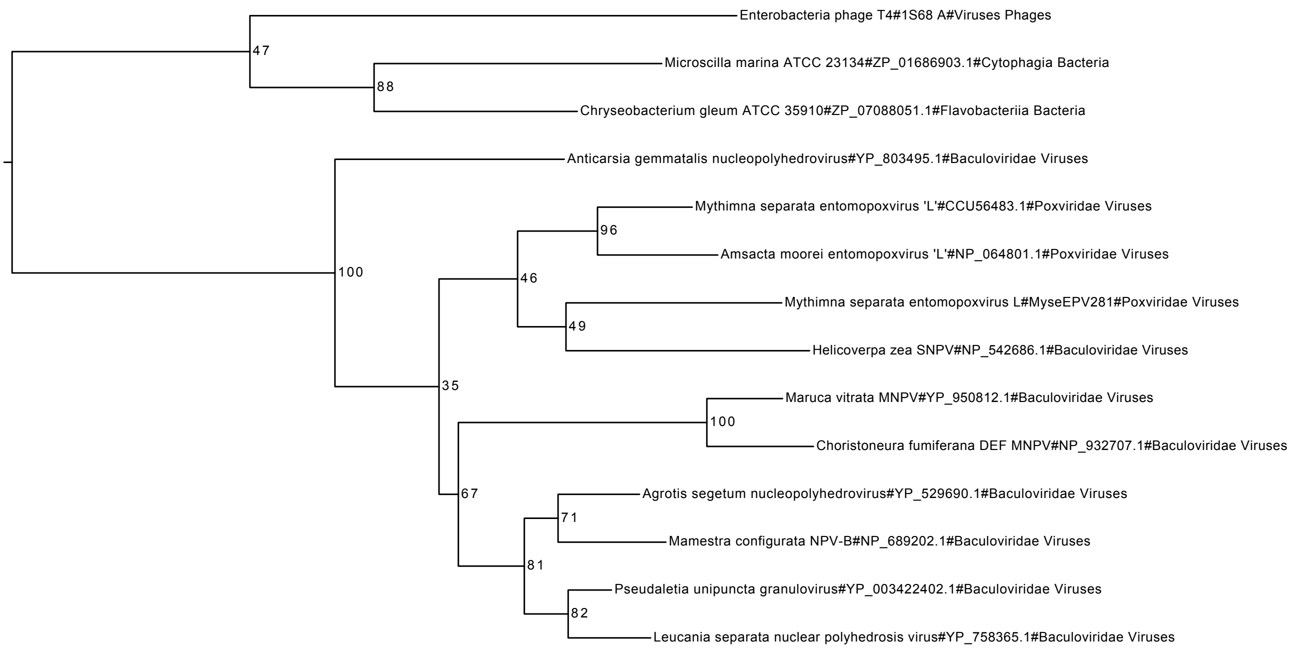

Figure S4. nla gene he65 (AcMNPV orf105) phylogeny. The tree was obtained from a maximum likelihood inference of the nla gene he65 (AcMNPV orf105) multiple amino acid alignments, including homologues found in baculoviruses and poxviruses, in other large DNA viruses and in cellular organisms. Support for nodes indicate maximum likelihood nonparametric bootstraps (100 replicates).

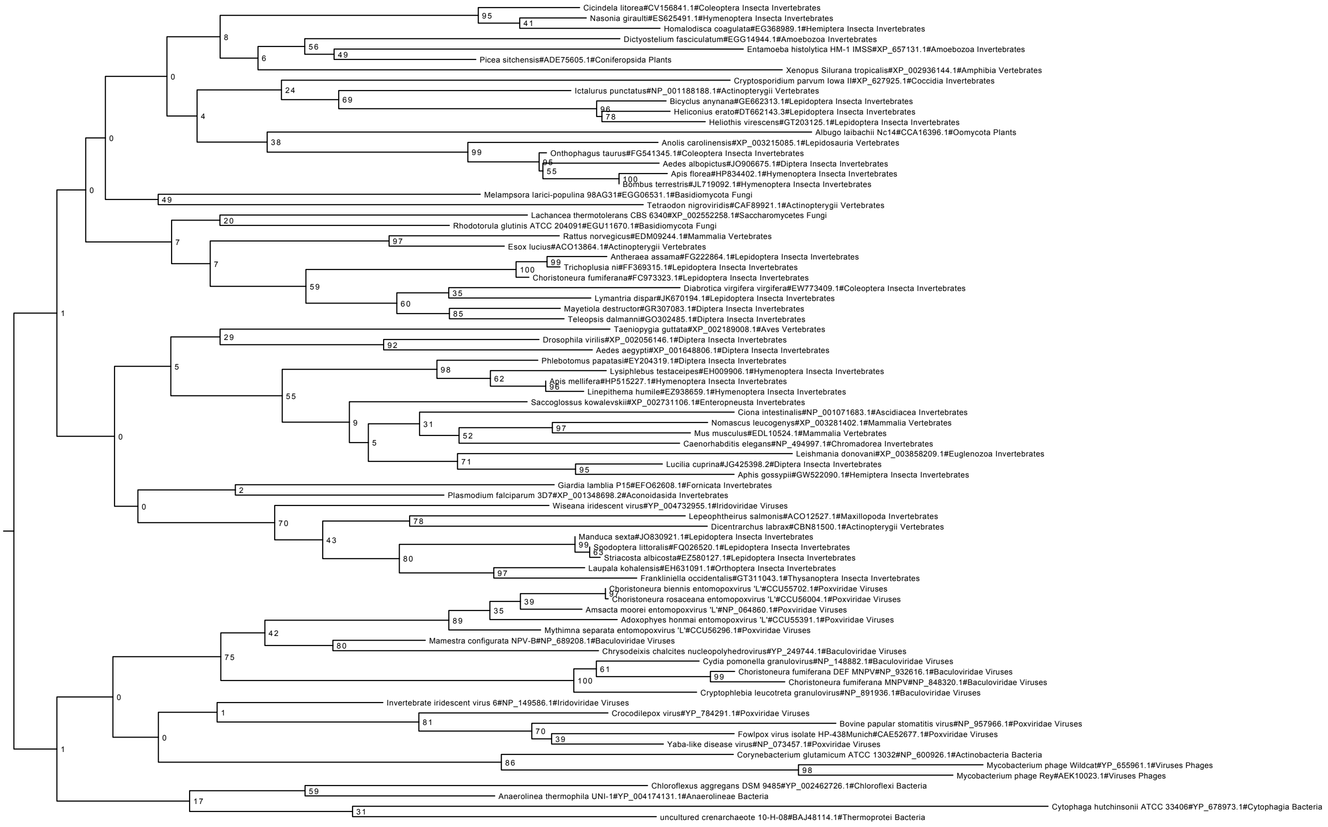

Figure S5. protein tyrosine phosphatase 2 (ptp-2) phylogeny. The tree was obtained from a maximum likelihood inference of the protein tyrosine phosphatase 2 (ptp-2) multiple amino acid alignments, including homologues found in baculoviruses and poxviruses, in other large DNA viruses and in cellular organisms. Support for nodes indicate maximum likelihood nonparametric bootstraps (100 replicates).

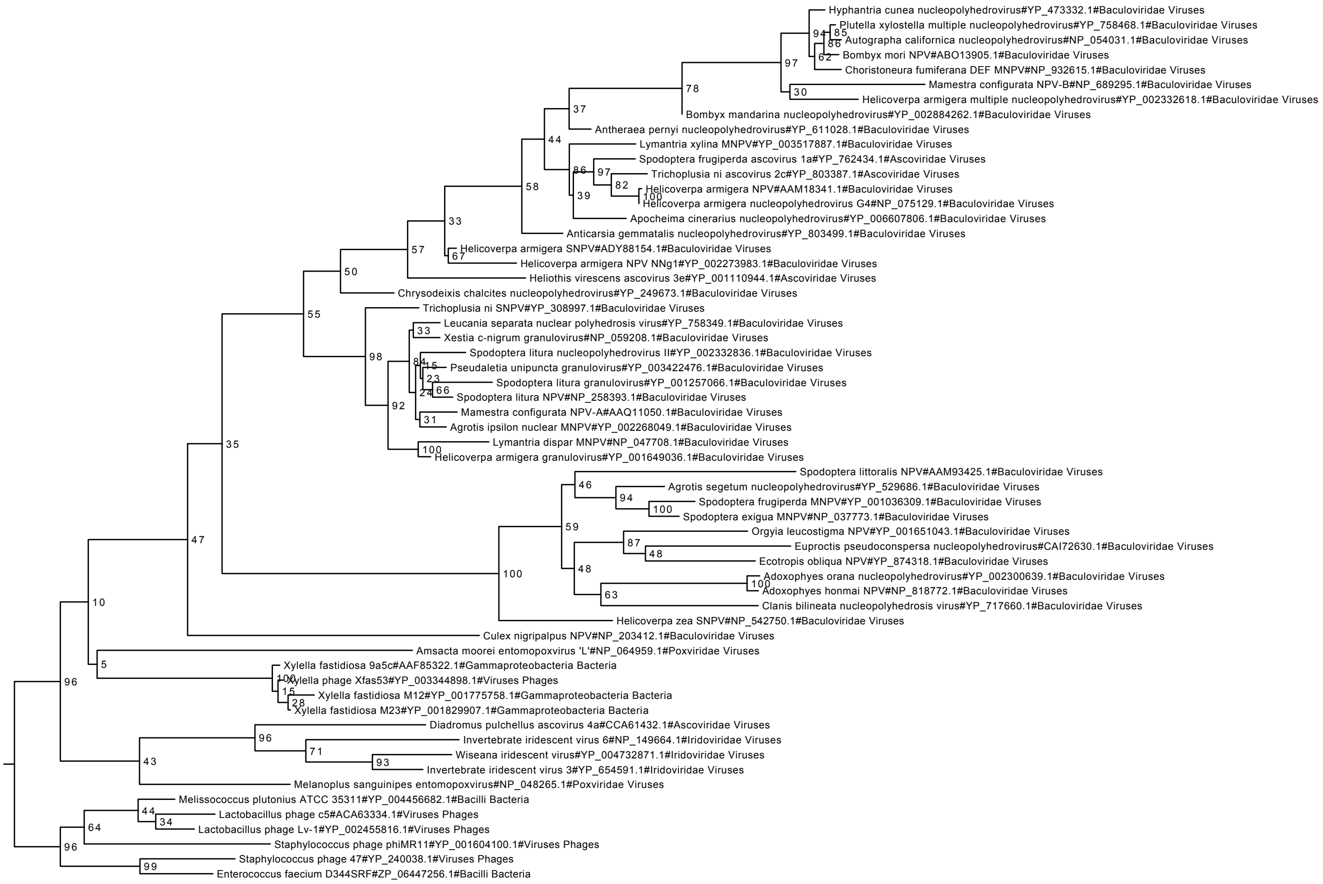

Figure S6. putative phage antirepressor phylogeny. The tree was obtained from a maximum likelihood inference of the putative phage antirepressor multiple amino acid alignments, including homologues found in baculoviruses and poxviruses, in other large DNA viruses and in cellular organisms. Support for nodes indicate maximum likelihood nonparametric bootstraps (100 replicates).

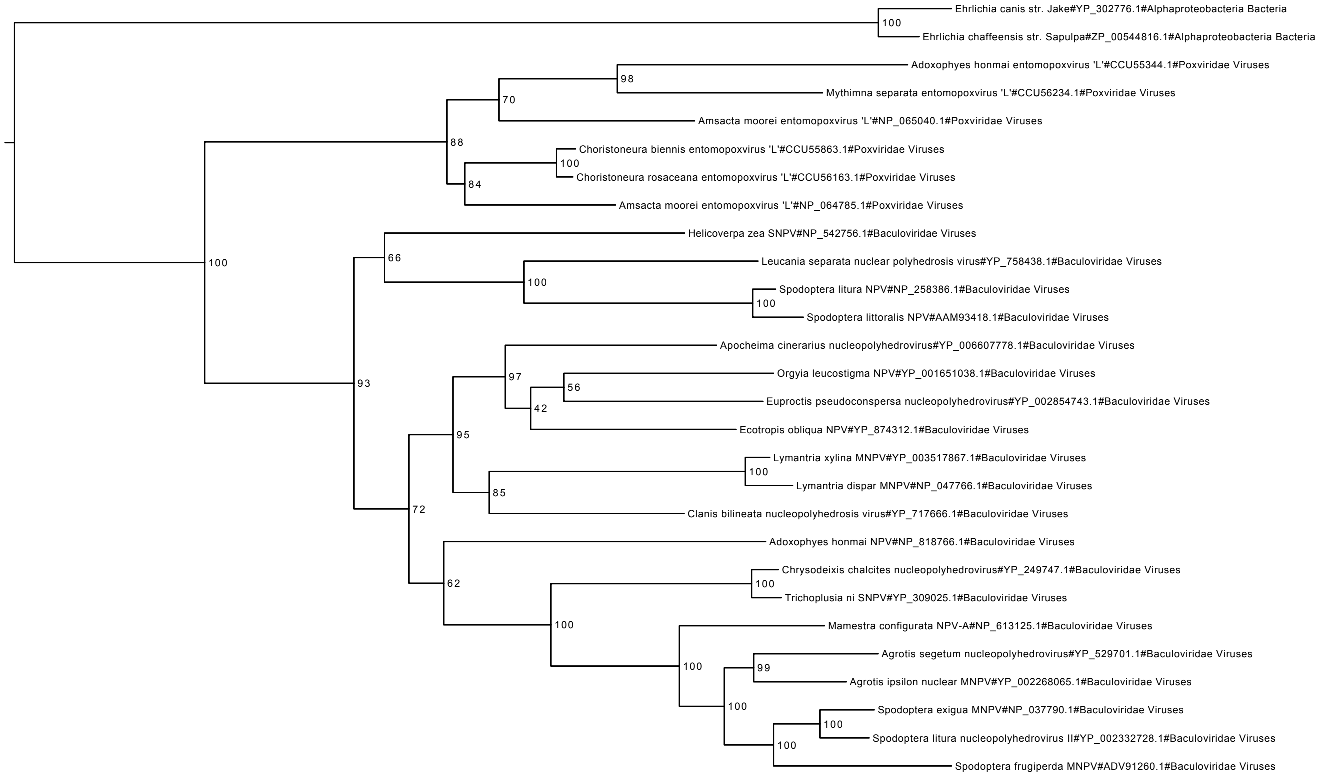

Figure S7. unknown LdMNPV orf129 phylogeny. The tree was obtained from a maximum likelihood inference of the unknown LdMNPV orf129 multiple amino acid alignments, including homologues found in baculoviruses and poxviruses, and in cellular organisms. Support for nodes indicate maximum likelihood nonparametric bootstraps (100 replicates).

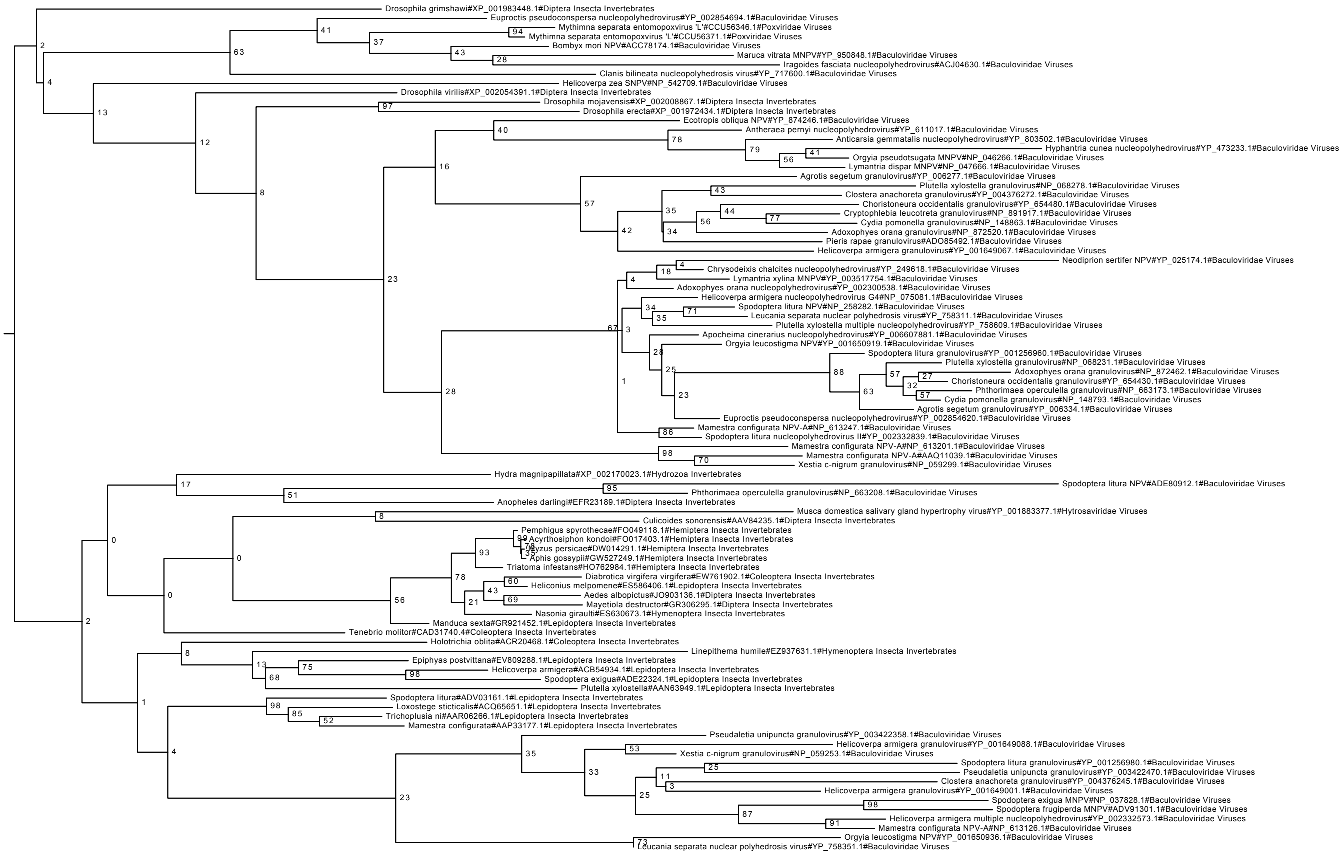

Figure S8. chitin binding protein (AcMNPV orf145) phylogeny. The tree was obtained from a maximum likelihood inference of the chitin binding protein (AcMNPV orf145) multiple amino acid alignments, including homologues found in baculoviruses and poxviruses, in other large DNA viruses and in cellular organisms. Support for nodes indicate maximum likelihood nonparametric bootstraps (100 replicates).

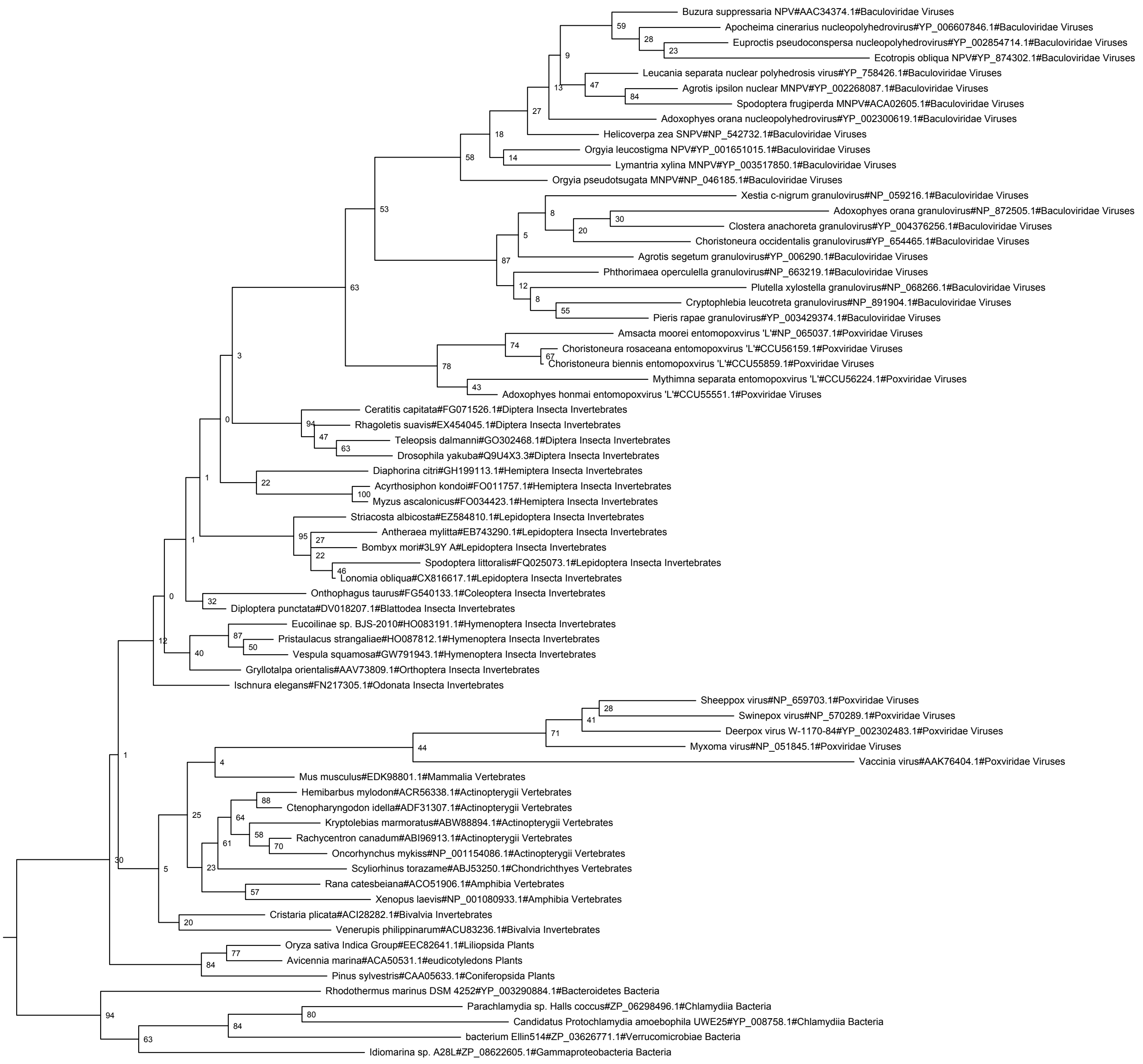

Figure S9. Cu/Zn superoxide dismutase phylogeny. The tree was obtained from a maximum likelihood inference of the Cu/Zn superoxide dismutase multiple amino acid alignments, including homologues found in baculoviruses and poxviruses, in other large DNA viruses and in cellular organisms. Support for nodes indicate maximum likelihood nonparametric bootstraps (100 replicates).

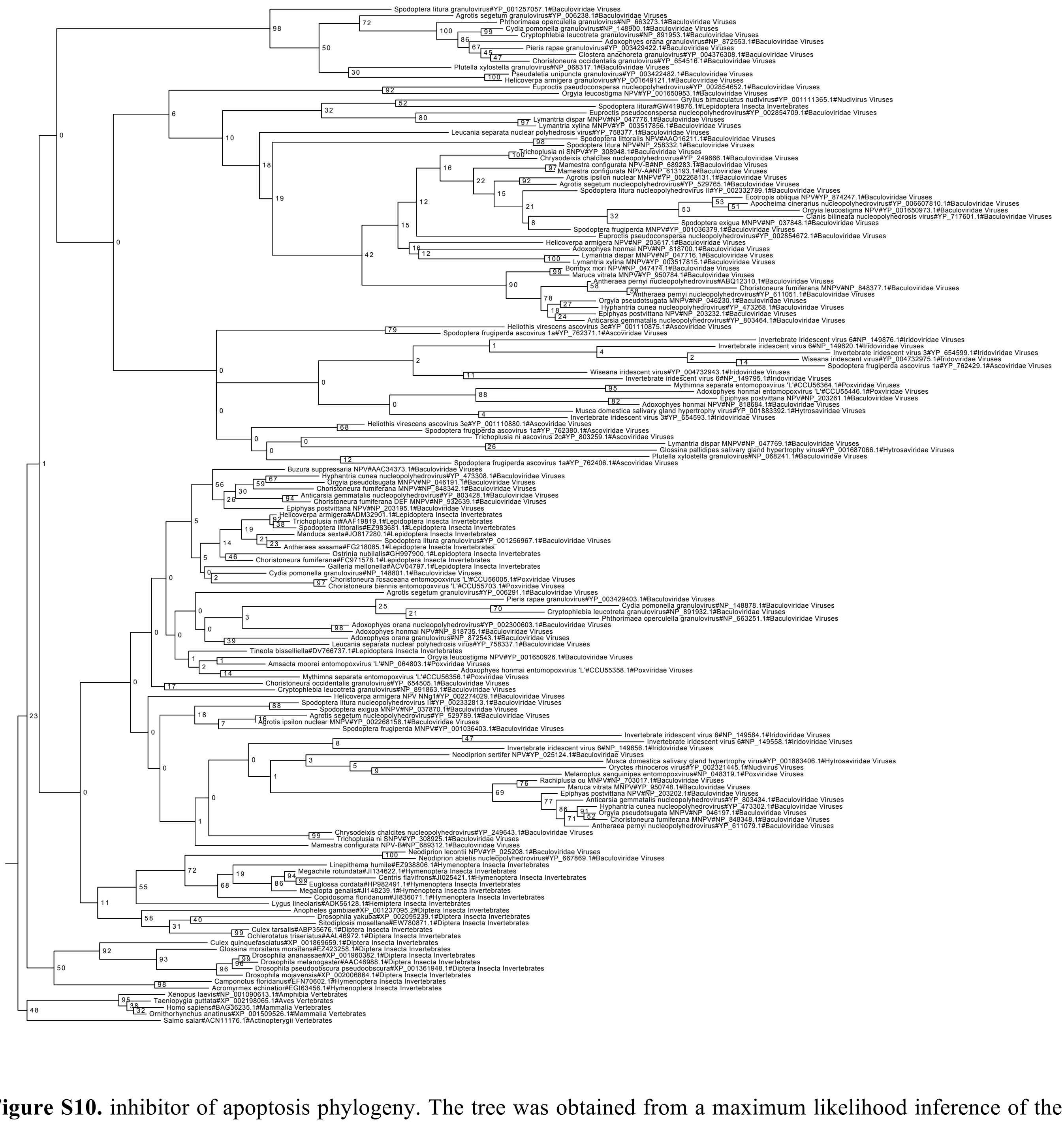

Figure S10. inhibitor of apoptosis phylogeny. The tree was obtained from a maximum likelihood inference of the inhibitor of apoptosis multiple amino acid alignments, including homologues found in baculoviruses and poxviruses, in other large DNA viruses and in cellular organisms. Support for nodes indicate maximum likelihood nonparametric bootstraps (100 replicates).

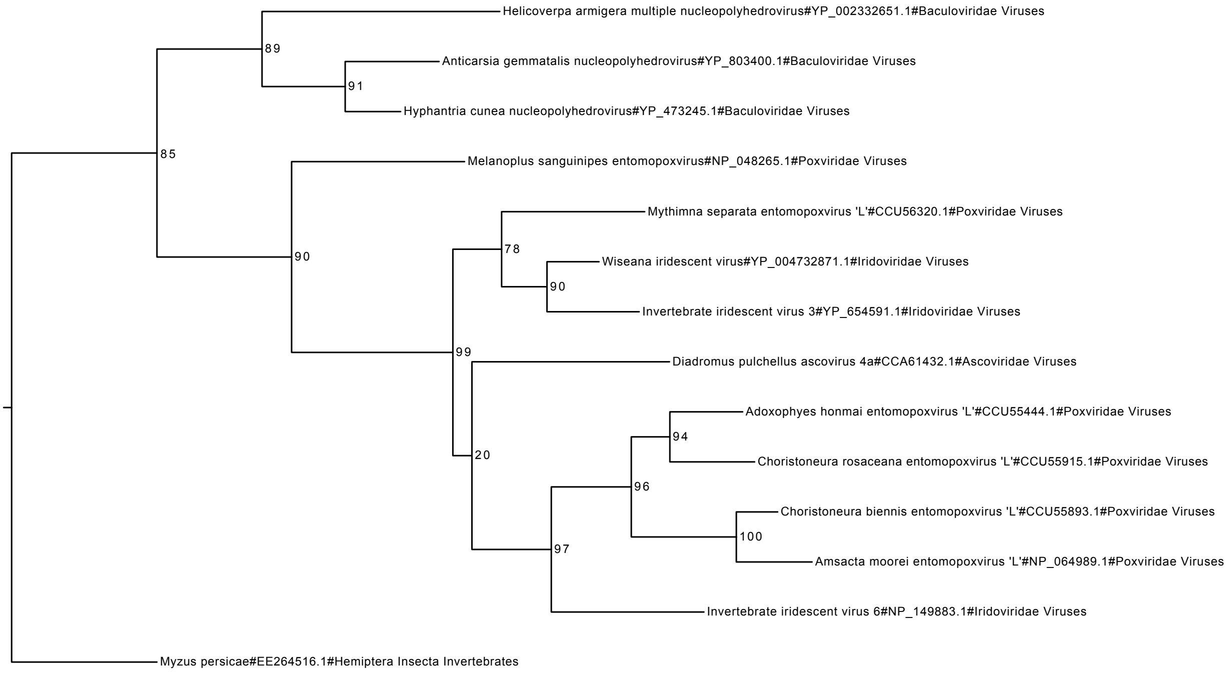

Figure S11. MTG motif gene family protein phylogeny. The tree was obtained from a maximum likelihood inference of the MTG motif gene family protein multiple amino acid alignments, including homologues found in baculoviruses and poxviruses, in other large DNA viruses and in cellular organisms. Support for nodes indicate maximum likelihood nonparametric bootstraps (100 replicates).

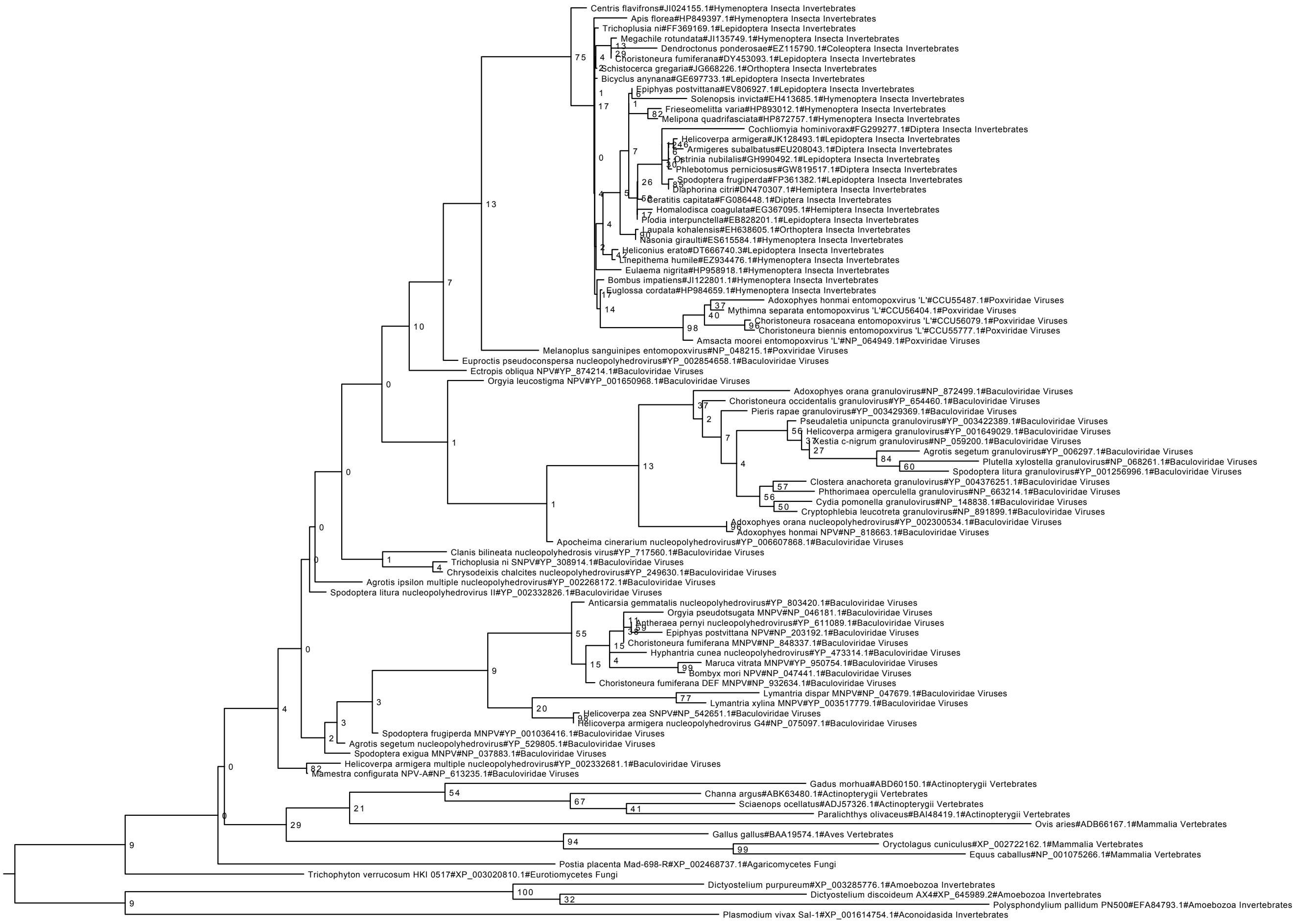

Figure S12. ubiquitin phylogeny. The tree was obtained from a maximum likelihood inference of the ubiquitin multiple amino acid alignments, including homologues found in baculoviruses and poxviruses, in other large DNA viruses and in cellular organisms. Support for nodes indicate maximum likelihood nonparametric bootstraps (100 replicates).

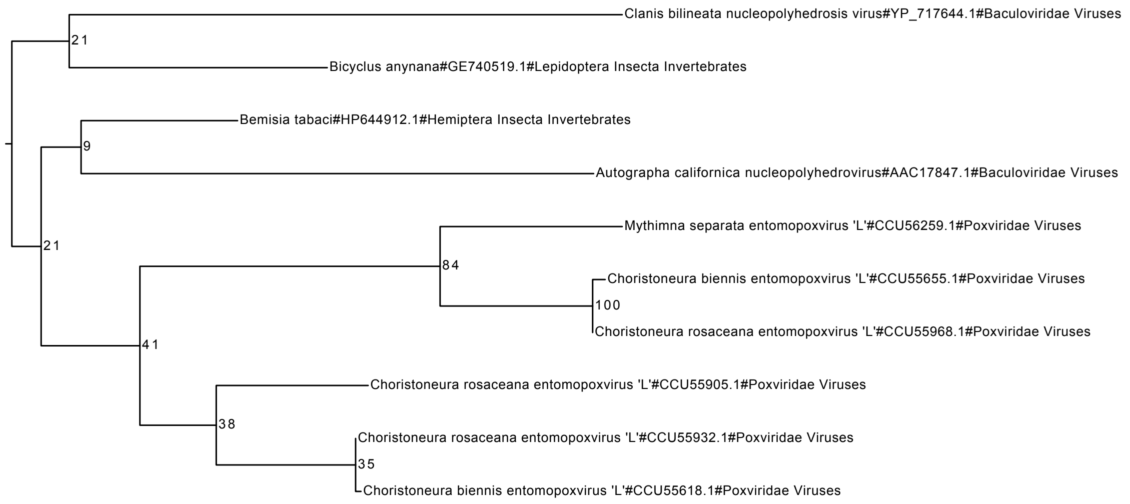

Figure S13. unknown AcMNPV orf7 phylogeny. The tree was obtained from a maximum likelihood inference of the unknown AcMNPV orf7 multiple amino acid alignments, including homologues found in baculoviruses and poxviruses, in other large DNA viruses and in cellular organisms. Support for nodes indicate maximum likelihood nonparametric bootstraps (100 replicates).

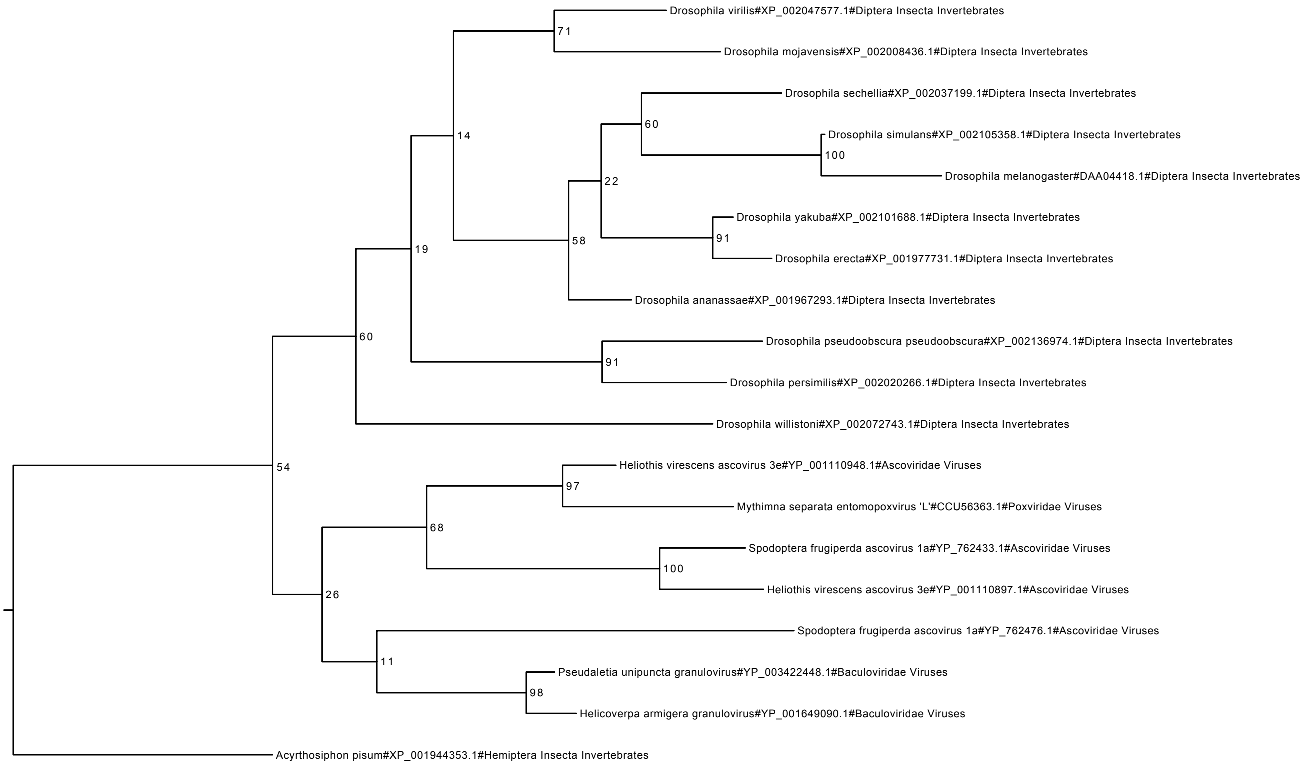

Figure S14. unknown XecnGV orf106 phylogeny. The tree was obtained from a maximum likelihood inference of the unknown XecnGV orf106 multiple amino acid alignments, including homologues found in baculoviruses and poxviruses, in other large DNA viruses and in cellular organisms. Support for nodes indicate maximum likelihood nonparametric bootstraps (100 replicates).

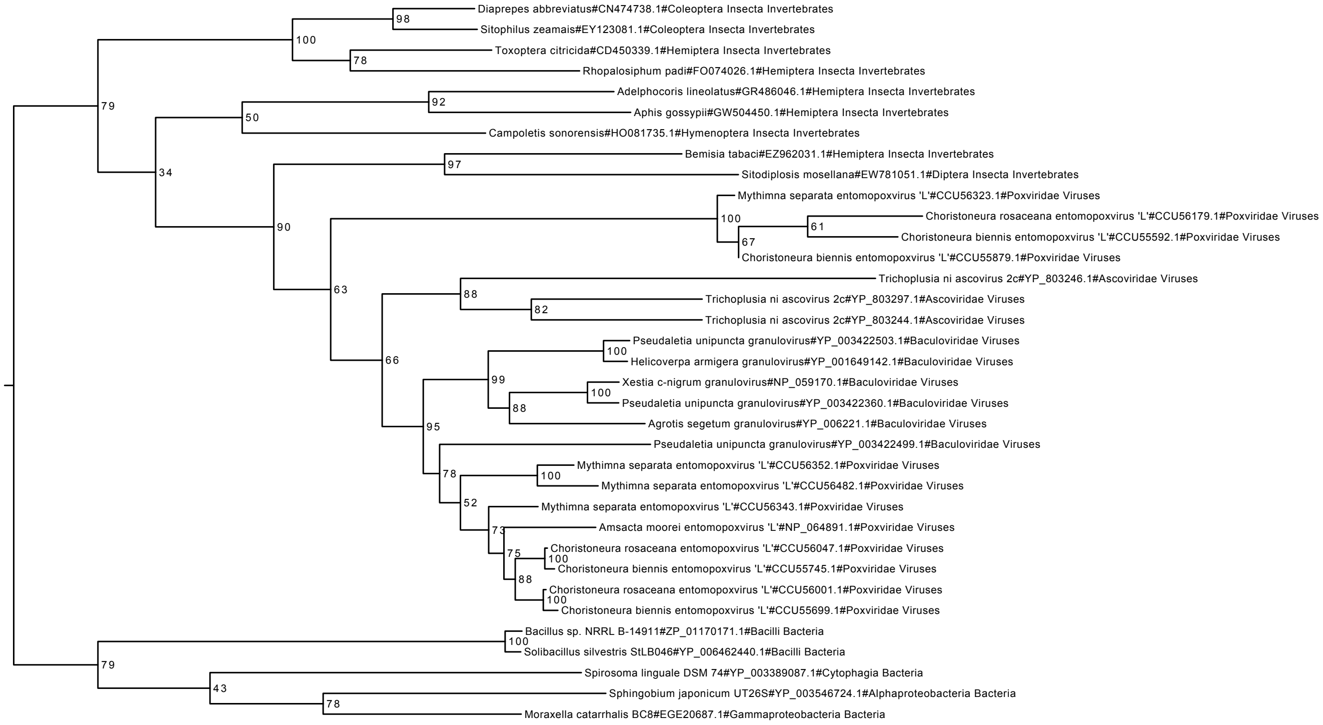

Figure S15. unknown XecnGV orf22 phylogeny. The tree was obtained from a maximum likelihood inference of the unknown XecnGV orf22 multiple amino acid alignments, including homologues found in baculoviruses and poxviruses, in other large DNA viruses and in cellular organisms. Support for nodes indicate maximum likelihood nonparametric bootstraps (100 replicates).

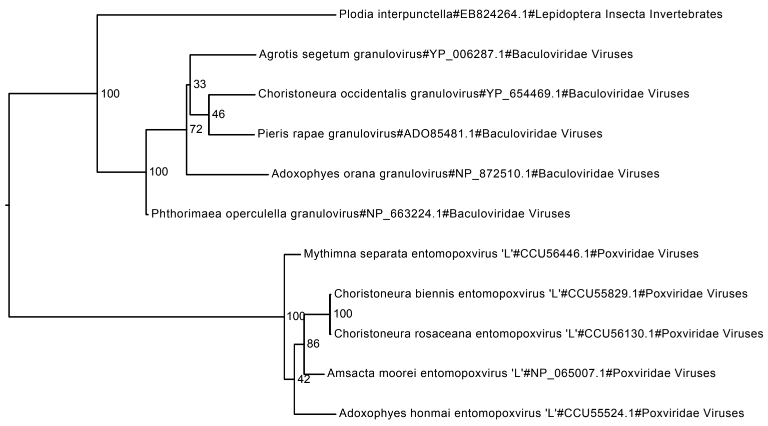

Figure S16. acetyltransferase phylogeny. The tree was obtained from a maximum likelihood inference of the acetyltransferase multiple amino acid alignments, including homologues found in baculoviruses and poxviruses, in other large DNA viruses and in cellular organisms. Support for nodes indicate maximum likelihood nonparametric bootstraps (100 replicates).

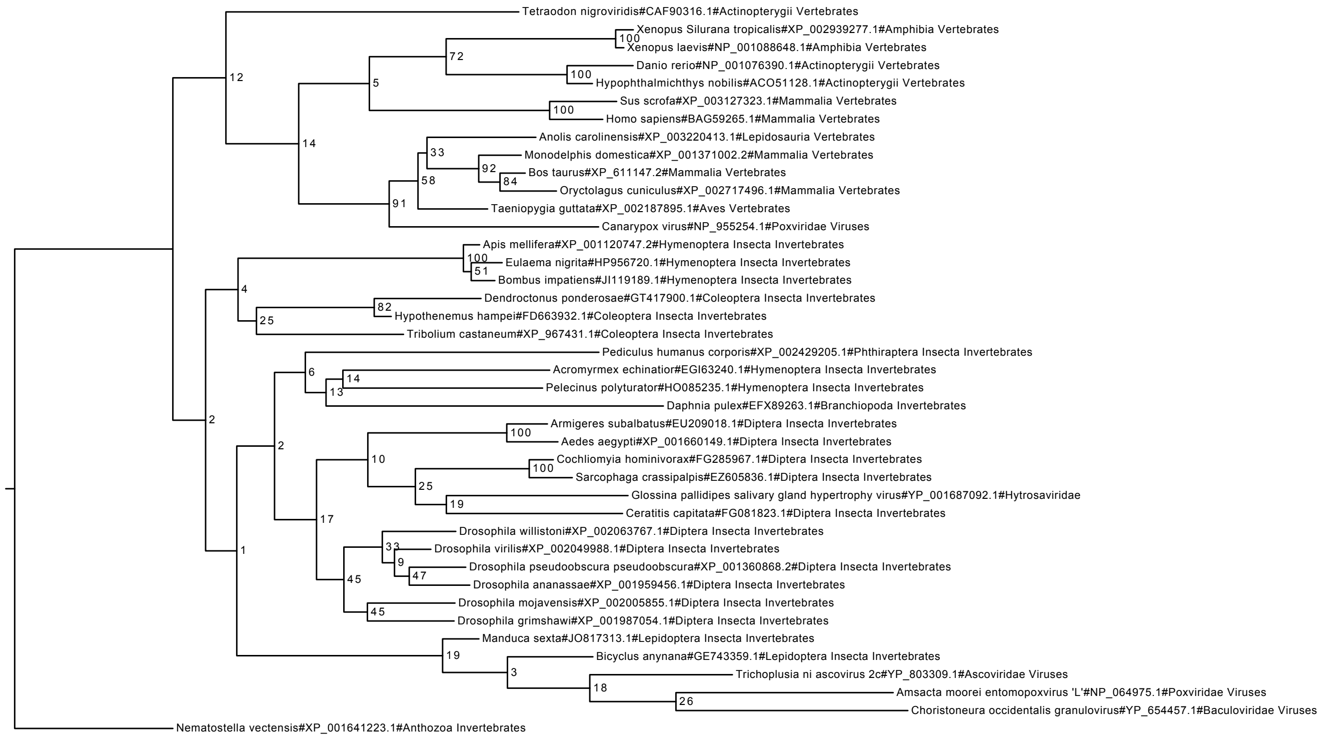

Figure S17. protein phosphatase 1, regulary subunit 15A phylogeny. The tree was obtained from a maximum likelihood inference of the protein phosphatase 1, regulary subunit 15A multiple amino acid alignments, including homologues found in baculoviruses and poxviruses, in other large DNA viruses and in cellular organisms. Support for nodes indicate maximum likelihood nonparametric bootstraps (100 replicates).

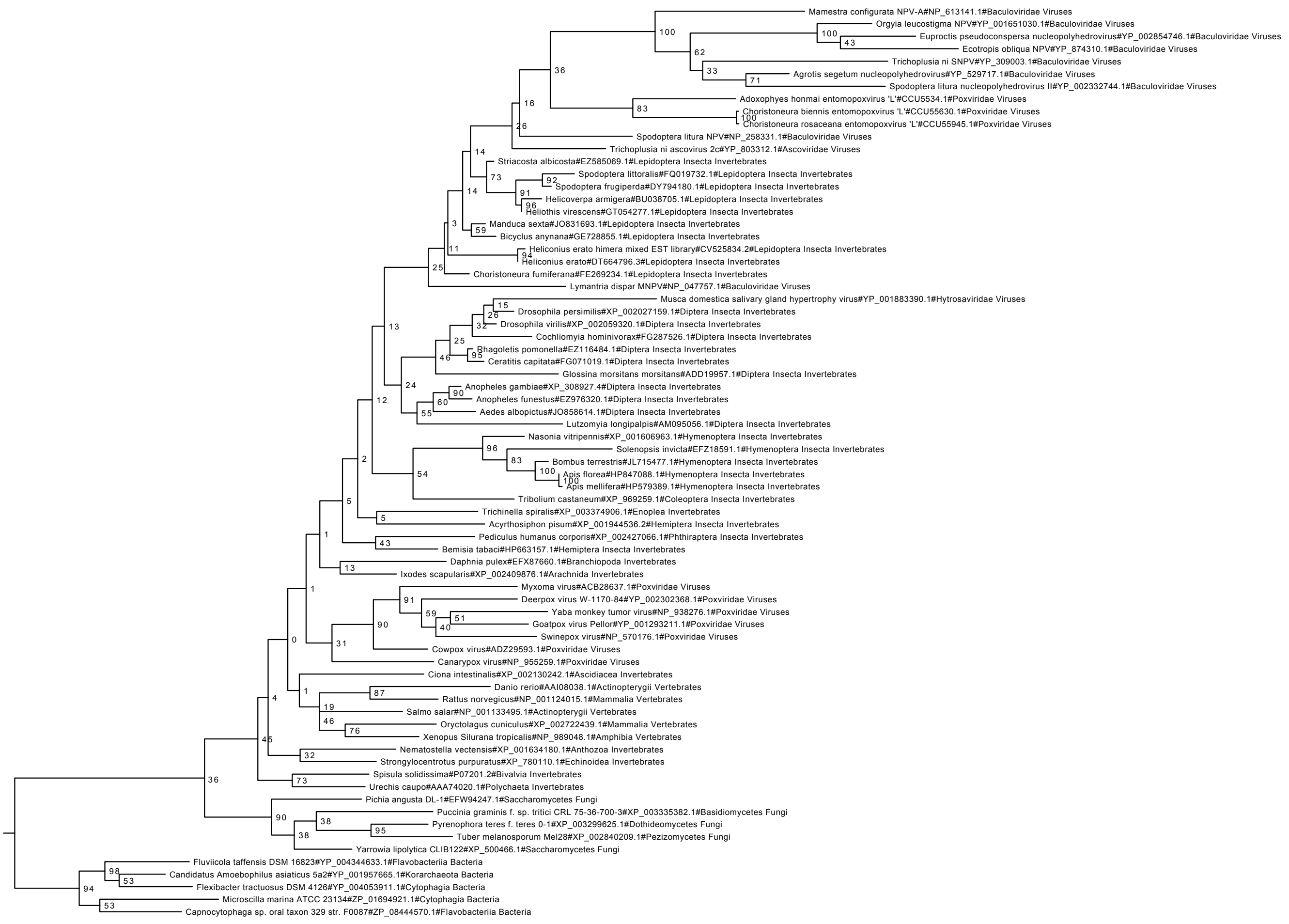

Figure S18. ribonucleotide reductase small subunit homolog phylogeny. The tree was obtained from a maximum likelihood inference of the ribonucleotide reductase small subunit homolog multiple amino acid alignments, including homologues found in baculoviruses and poxviruses, in other large DNA viruses and in cellular organisms. Support for nodes indicate maximum likelihood nonparametric bootstraps (100 replicates).

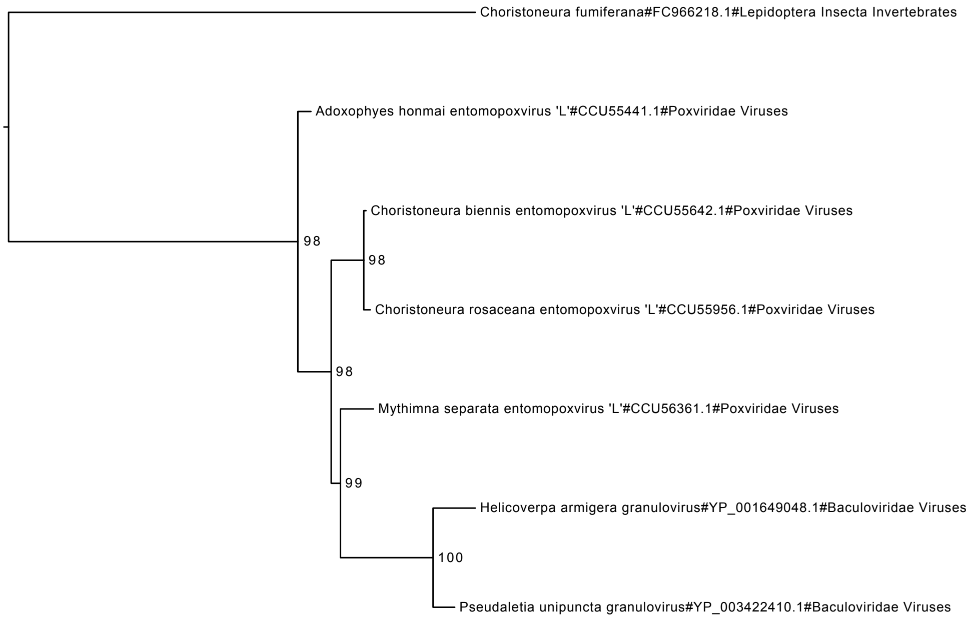

Figure S19. unknown XecnGV orf72 phylogeny. The tree was obtained from a maximum likelihood inference of the unknown XecnGV orf72 multiple amino acid alignments, including homologues found in baculoviruses and poxviruses, and in cellular organisms. Support for nodes indicate maximum likelihood nonparametric bootstraps (100 replicates).

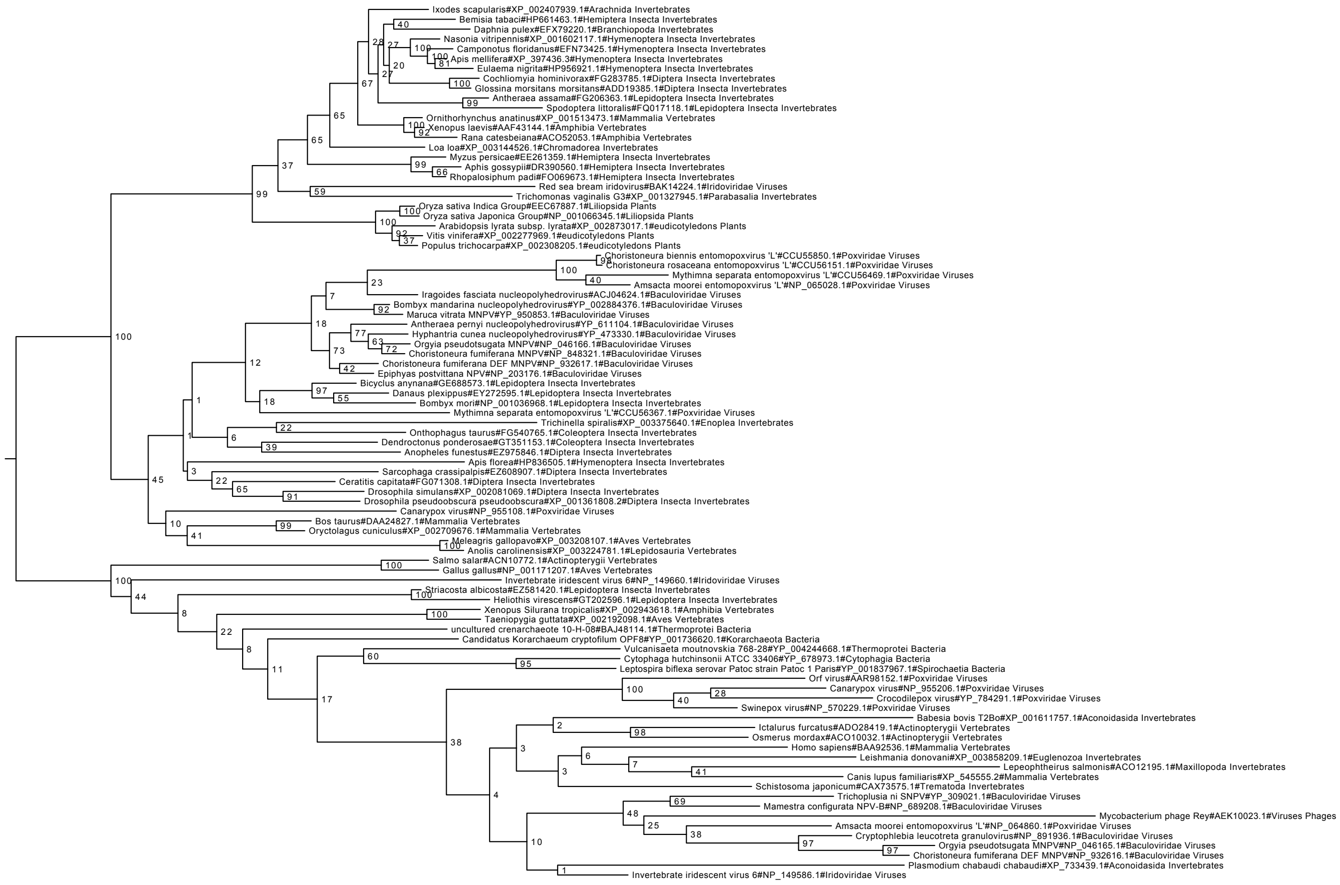

Figure S20. protein tyrosine phosphatase 1 (ptp-1) phylogeny. The tree was obtained from a maximum likelihood inference of the protein tyrosine phosphatase 1 (ptp-1) multiple amino acid alignments, including homologues found in baculoviruses and poxviruses, and in cellular organisms. Support for nodes indicate maximum likelihood nonparametric bootstraps (100 replicates).

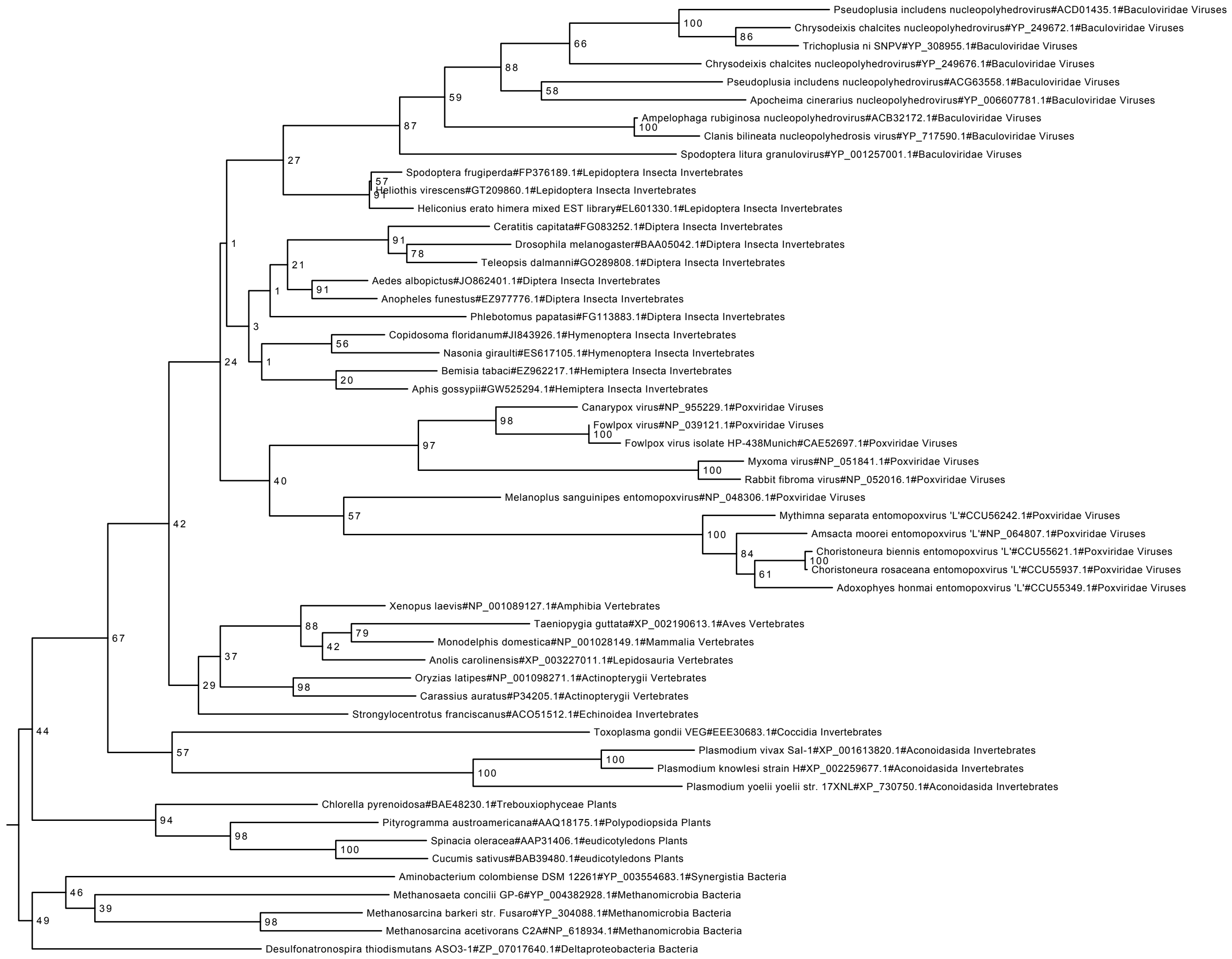

Figure S21. DNA photolyase phylogeny. The tree was obtained from a maximum likelihood inference of the DNA photolyase multiple amino acid alignments, including homologues found in baculoviruses and poxviruses, and in cellular organisms. Support for nodes indicate maximum likelihood nonparametric bootstraps (100 replicates).

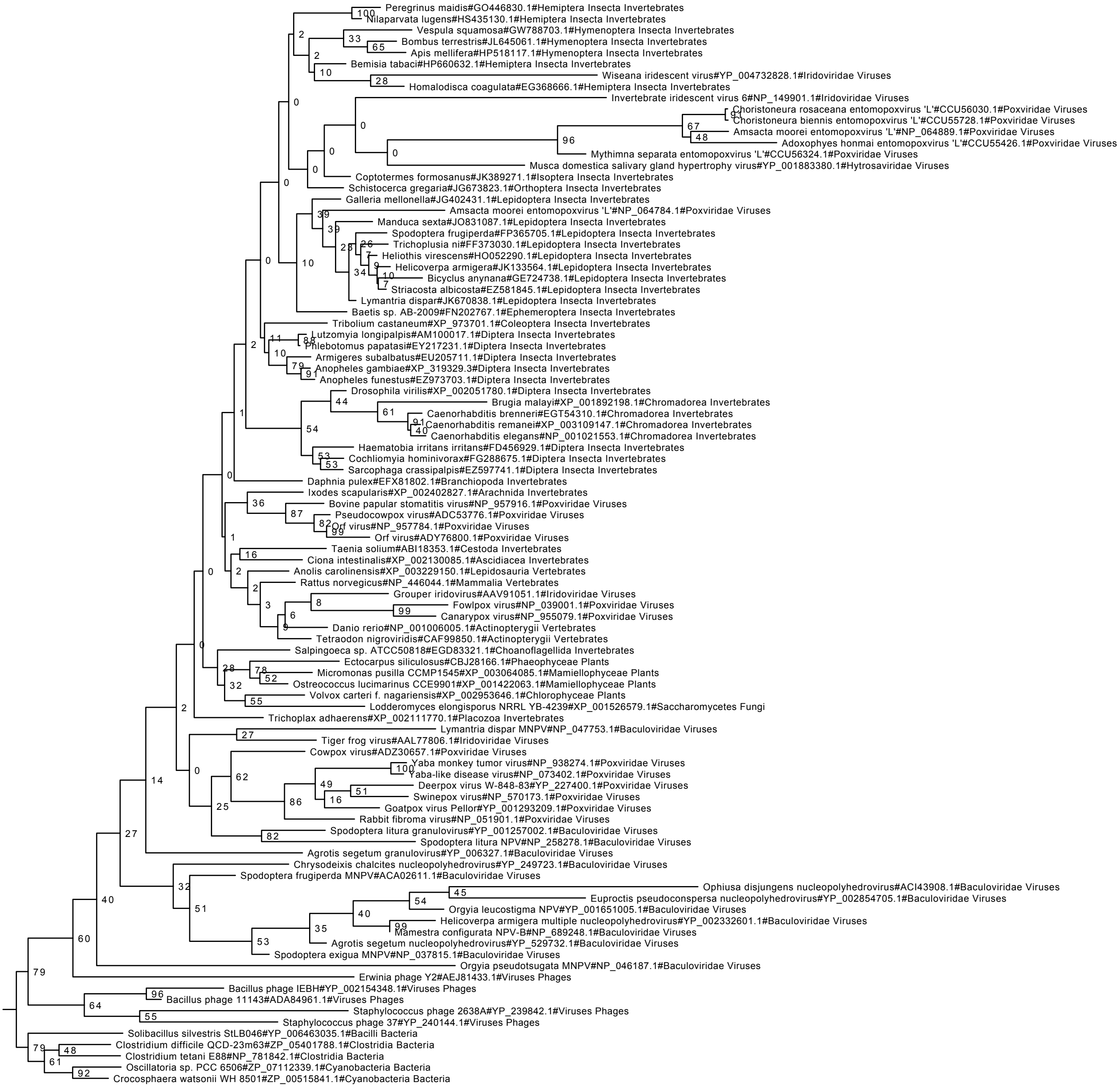

Figure S22. dUTPase phylogeny. The tree was obtained from a maximum likelihood inference of the dUTPase multiple amino acid alignments, including homologues found in baculoviruses and poxviruses, in other large DNA viruses and in cellular organisms. Support for nodes indicate maximum likelihood nonparametric bootstraps (100 replicates).

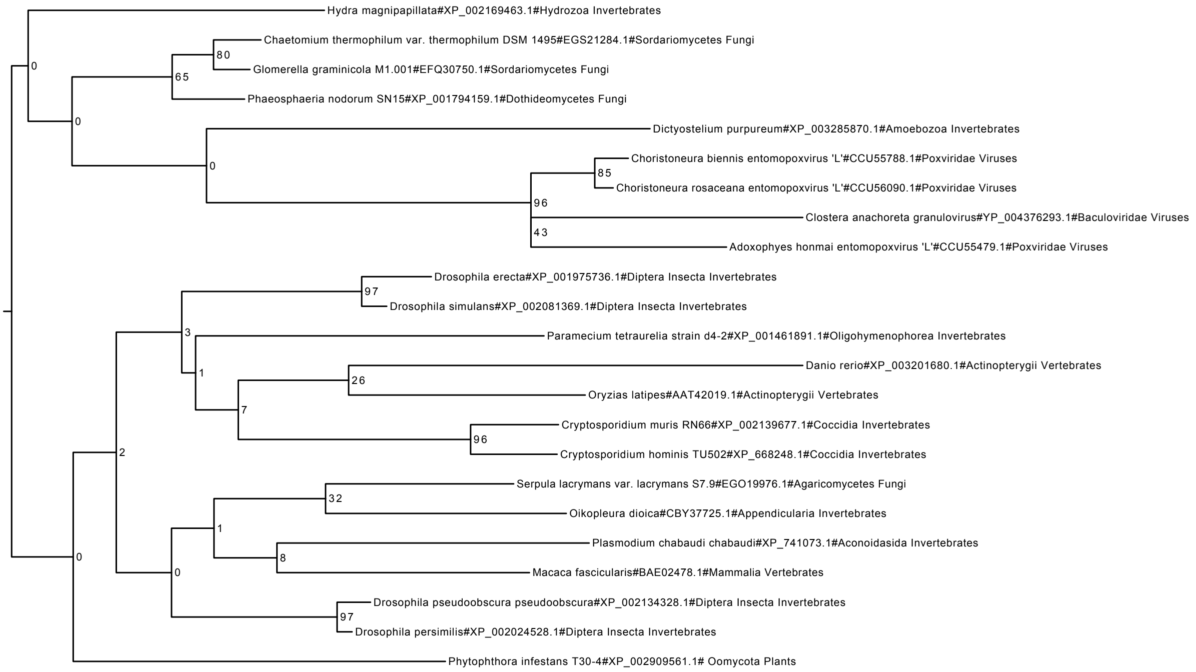

Figure S23. unknown ClanGV orf085 phylogeny. The tree was obtained from a maximum likelihood inference of the unknown ClanGV orf085 multiple amino acid alignments, including homologues found in baculoviruses and poxviruses, and in cellular organisms. Support for nodes indicate maximum likelihood nonparametric bootstraps (100 replicates).

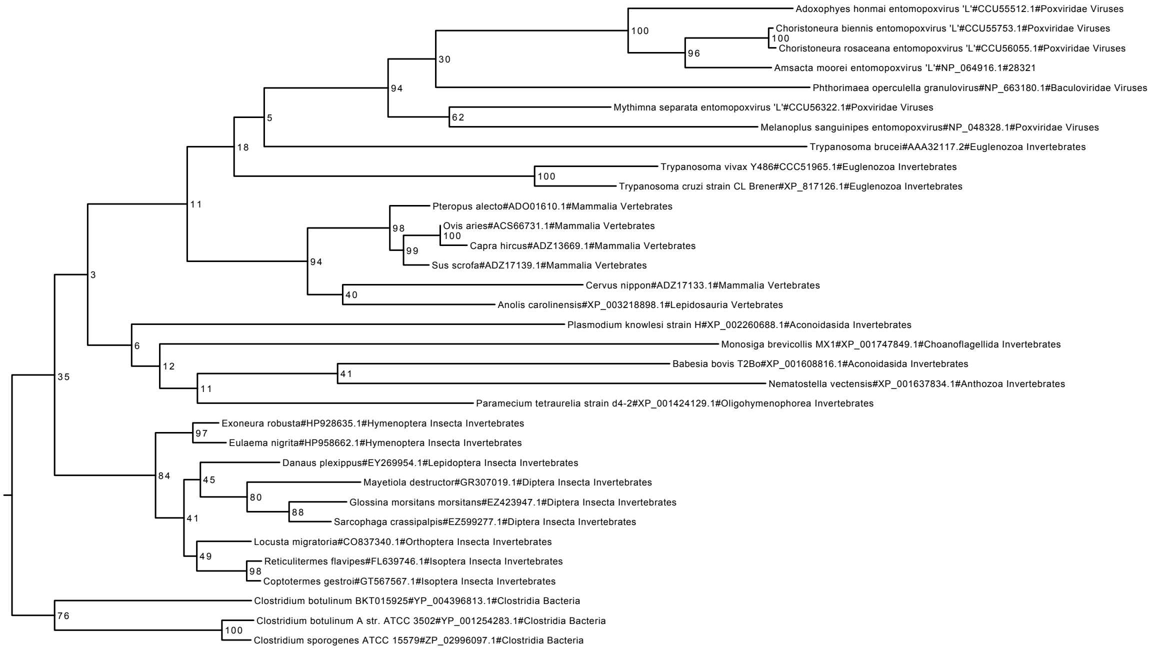

Figure S24. leucine rich gene family protein phylogeny. The tree was obtained from a maximum likelihood inference of the leucine rich gene family protein multiple amino acid alignments, including homologues found in baculoviruses and poxviruses, and in cellular organisms. Support for nodes indicate maximum likelihood nonparametric bootstraps (100 replicates).

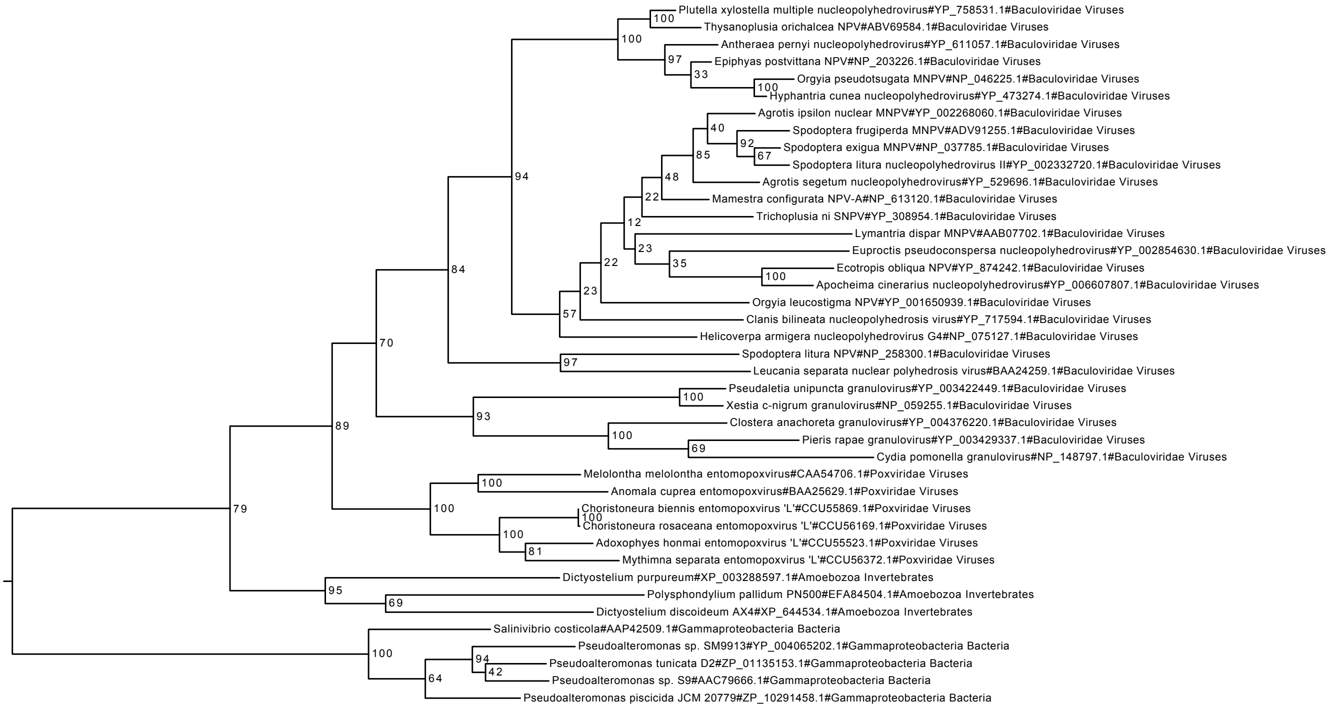

Figure S25. fusolin/spindlin/gp37 (AcMNPV orf64) phylogeny. The tree was obtained from a maximum likelihood inference of the fusolin/spindlin/gp37 (AcMNPV orf64) multiple amino acid alignments, including homologues found in baculoviruses and poxviruses, and in cellular organisms. Support for nodes indicate maximum likelihood nonparametric bootstraps (100 replicates).

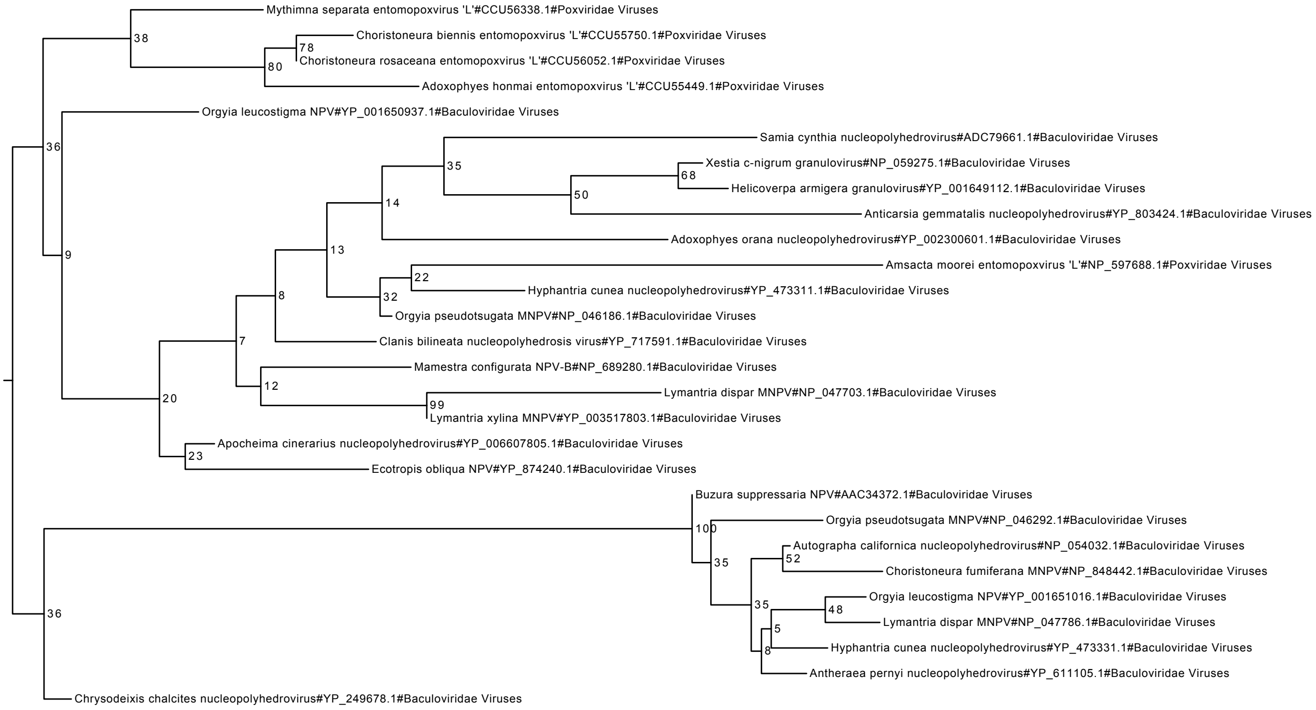

Figure S26. conotoxin-like protein phylogeny. The tree was obtained from a maximum likelihood inference of the conotoxin-like protein multiple amino acid alignments, including homologues found in baculoviruses and poxviruses. Support for nodes indicate maximum likelihood nonparametric bootstraps (100 replicates).

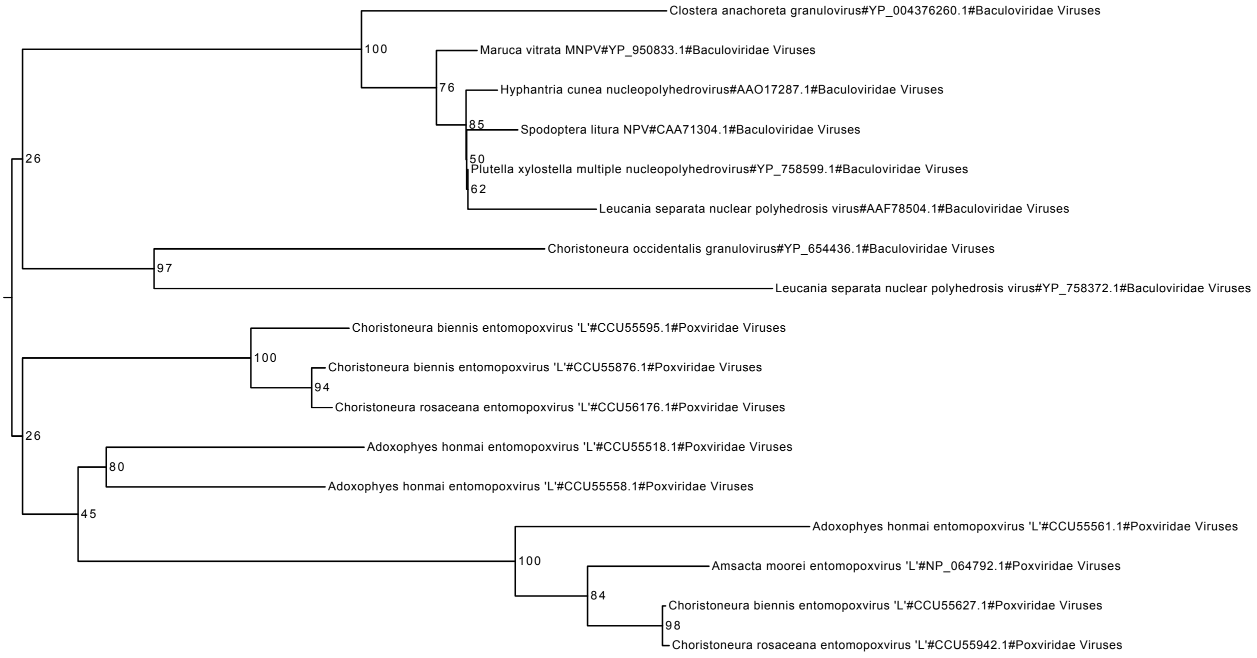

Figure S27. p35/p49 apoptosis inhibitor phylogeny. The tree was obtained from a maximum likelihood inference of the p35/p49 apoptosis inhibitor multiple amino acid alignments, including homologues found in baculoviruses and poxviruses. Support for nodes indicate maximum likelihood nonparametric bootstraps (100 replicates).

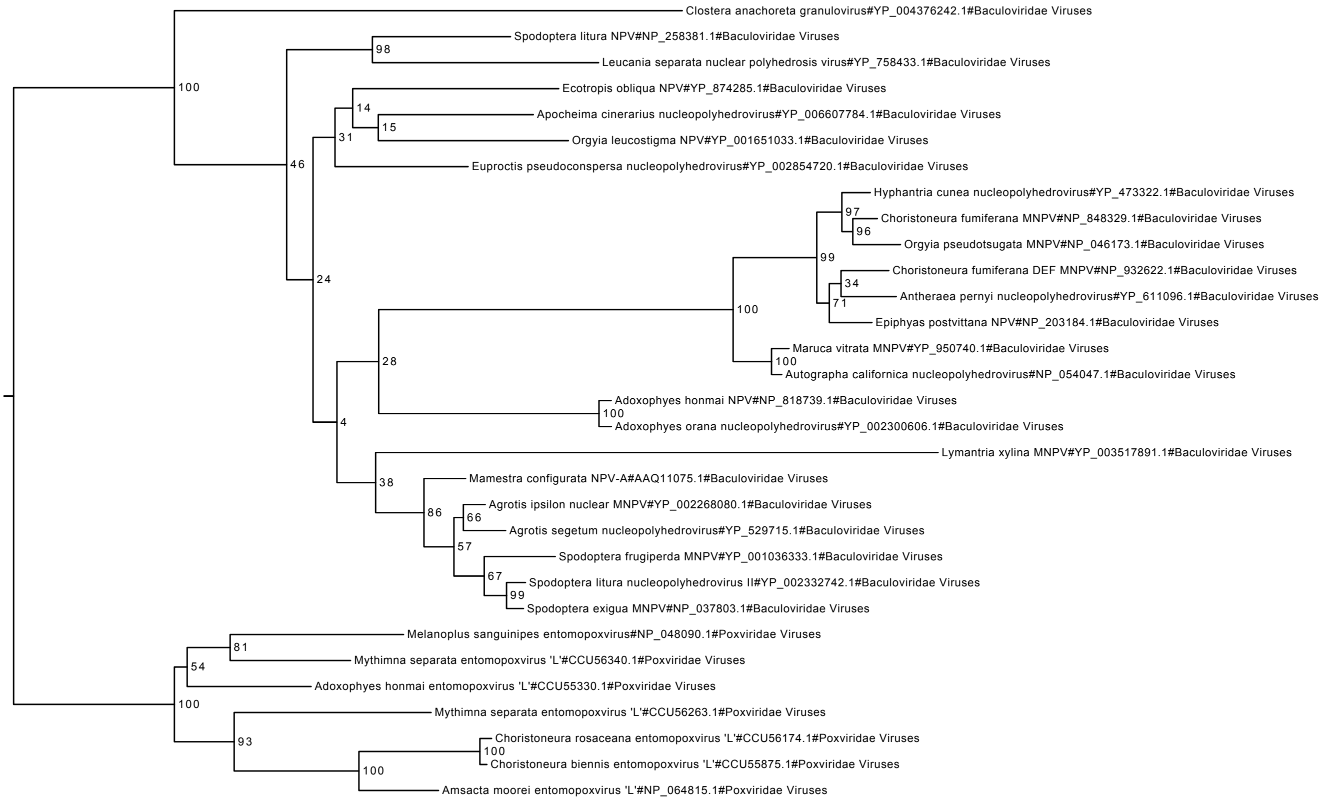

Figure S28. unknown AcMNPV orf18 phylogeny. The tree was obtained from a maximum likelihood inference of the unknown AcMNPV orf18 multiple amino acid alignments, including homologues found in baculoviruses and poxviruses. Support for nodes indicate maximum likelihood nonparametric bootstraps (100 replicates).

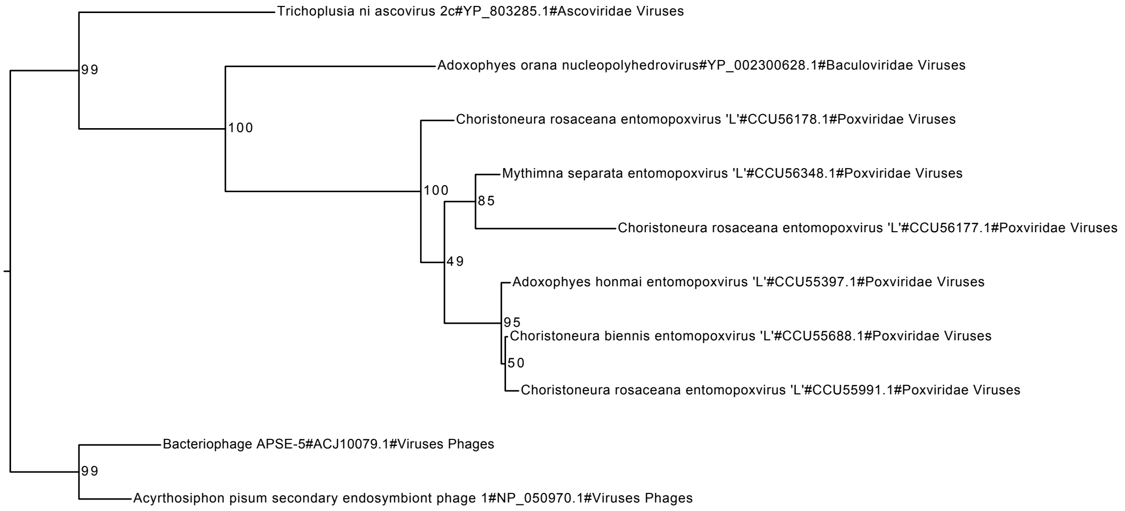

Figure S29. unknown AdorNPV orf110 phylogeny. The tree was obtained from a maximum likelihood inference of the unknown AdorNPV orf110 multiple amino acid alignments, including homologues found in baculoviruses and poxviruses, and in other large DNA viruses. Support for nodes indicate maximum likelihood nonparametric bootstraps (100 replicates).
